# ASY1 acts as a dosage-dependent antagonist of telomere-led recombination and mediates crossover interference in *Arabidopsis*

**DOI:** 10.1073/pnas.1921055117

**Published:** 2020-06-04

**Authors:** Christophe Lambing, Pallas C. Kuo, Andrew J. Tock, Stephanie D. Topp, Ian R. Henderson

**Affiliations:** ^a^Department of Plant Sciences, University of Cambridge, CB2 3EA Cambridge, United Kingdom

**Keywords:** meiosis, crossover, axis, ASY1, interference

## Abstract

During meiosis, interhomolog recombination produces crossovers and noncrossovers to create genetic diversity. Meiotic recombination frequency varies at multiple scales, with high subtelomeric recombination and suppressed centromeric recombination typical in many eukaryotes. During recombination, sister chromatids are tethered as loops to a polymerized chromosome axis, which, in plants, includes the ASY1 HORMA domain protein and REC8–cohesin complexes. Using chromatin immunoprecipitation, we show an ascending telomere-to-centromere gradient of ASY1 enrichment, which correlates strongly with REC8–cohesin ChIP-seq data. We mapped crossovers genome-wide in the absence of ASY1 and observe that telomere-led recombination becomes dominant. Surprisingly, *asy1/+* heterozygotes also remodel crossovers toward subtelomeric regions at the expense of the pericentromeres. Telomeric recombination increases in *asy1/+* occur in distal regions where ASY1 and REC8 ChIP enrichment are lowest in wild type. In wild type, the majority of crossovers show interference, meaning that they are more widely spaced along the chromosomes than expected by chance. To measure interference, we analyzed double crossover distances, MLH1 foci, and fluorescent pollen tetrads. Interestingly, while crossover interference is normal in *asy1/+*, it is undetectable in *asy1* mutants, indicating that ASY1 is required to mediate crossover interference. Together, this is consistent with ASY1 antagonizing telomere-led recombination and promoting spaced crossover formation along the chromosomes via interference. These findings provide insight into the role of the meiotic axis in patterning recombination frequency within plant genomes.

## Significance

Meiosis is fundamental to eukaryotic reproduction and shapes patterns of genetic variation. Meiotic recombination is also a vital tool during crop improvement, which allows introgression of wild variation into agricultural strains. Despite this, the levels and distributions of crossovers along chromosomes can limit breeding. For example, many crops show highly skewed crossover distributions toward the telomeres. This can lead to the problem of linkage drag when variation within nonrecombining regions is selected. Our findings demonstrate how gene dosage of key components of the meiotic chromosome axis can be used to remodel the recombination landscape. Therefore, modifying *ASY1* and *ASY3* gene dosage in crop species may provide a strategy to change recombination patterns or levels in order to accelerate strain improvement.

Meiosis is a specialized cell division that increases genetic diversity in populations (1, 2). Meiosis halves the chromosome number to produce haploid gametes via a single round of DNA replication and two rounds of chromosome segregation (1, 3). During prophase I of meiosis, homologous chromosomes undergo DNA double-strand breaks (DSBs) that can be repaired using an interhomolog pathway, which may result in crossovers or non–crossovers (1, 3). In plants, meiotic DSBs are formed via a topoisomerase-VI–like complex containing SPO11-1, SPO11-2, and MTOPVIB (4). Meiotic DSBs are resected to form 3′-overhanging single-stranded DNA (ssDNA), which is bound by the RecA homologs RAD51 and DMC1 that promote strand invasion of a homolog (1, 3). A set of pro–crossover factors, termed the ZMM pathway, act to protect interhomolog strand invasion events from antirecombination pathways (3). Class I crossover events generated via the ZMM pathway are more widely spaced along the chromosomes than expected by chance, which is known as interference (5). A minority of crossovers are generated by the Class II repair pathways in wild type, which do not show interference (3).

Homologous chromosomes associate with a specialized axis structure during meiosis, which is conserved across eukaryotes and is required for efficient and accurate interhomolog recombination (6). Following S-phase, replicated sister chromatids are associated via cohesin complexes containing the meiosis-specific kleisin REC8 (7, 8). Immunostaining of REC8 during prophase I reveals a linear axis, to which the chromatin is attached (6). In addition to REC8–cohesin, major components of the plant meiotic chromosome axis include the HORMA domain protein ASY1 and the coiled-coil proteins ASY3 and ASY4 (9–12). In this configuration, coaligned chromatin loops project laterally from the axis, resembling mitotic lampbrush configurations, although with a juxtaposed homolog (6). The tethered-loop axis model proposes that meiotic DSBs are generated on the chromatin loops that become tethered to the axis during interhomolog repair (6, 13). Axis-localized HORMA domain proteins are required during meiosis to promote homolog pairing, DSB repair, and synaptonemal complex (SC) assembly (14–19). However, there are also important differences in the function of meiotic HORMA proteins between species. For example, mouse HORMAD1, budding yeast Hop1, and *Caenorhabditis elegans* HTP-3, but not *Arabidopsis* ASY1, are required for meiotic DSB formation (9, 15, 20–22). In late prophase I, the axis is remodeled, which is associated with depletion of HORMA proteins and loading of transverse filament SC proteins, including ZYP1a and ZYP1b (18, 23).

Genome-wide analyses have revealed that meiotic DSB and crossover frequency are highly variable between the telomeres and centromeres of plant chromosomes (24–29). For example, the centromeres and surrounding repetitive sequences (pericentromeric heterochromatin) are frequently suppressed for meiotic recombination (24–29). High meiotic crossover levels are typically observed in distal subtelomeric regions, which also tend to have higher gene density (24–29). However, the factors and mechanisms that shape the meiotic recombination landscape along chromosomes remain incompletely understood. To investigate the role of the axis during meiosis, we mapped ASY1 enrichment throughout the *Arabidopsis* genome using chromatin immunoprecipitation sequencing (ChIP-seq). We observe an ascending ASY1 gradient from the telomere to the centromere, which correlates positively with REC8 ChIP-seq data (30). We mapped crossovers genome-wide in *asy1* mutants and observe that recombination becomes telomere-led, likely reflecting telomere pairing observed early in prophase I (31). We show that *asy1/+* heterozygotes maintain crossover numbers but remodel recombination frequency toward the telomeres at the expense of the pericentromeres. The zone of telomere-led recombination in *asy1* and *asy1/+* corresponds to distal regions of the chromosomes with lowest ASY1 and REC8 ChIP-seq enrichment in wild type. Through analysis of double crossover distances, fluorescent pollen tetrads, and MLH1 foci, we show that crossover interference is normal in *asy1/+* heterozygotes, but is undetectable in *asy1* homozygotes. Together, our data show that ASY1 exerts a major effect on the crossover landscape via mediating interference and acting as a gene dosage-dependent antagonist of telomere-led recombination.

## Results

### Telomere–Centromere Gradients of ASY1 and REC8 ChIP-Seq Enrichment.

**Fig. 1.**
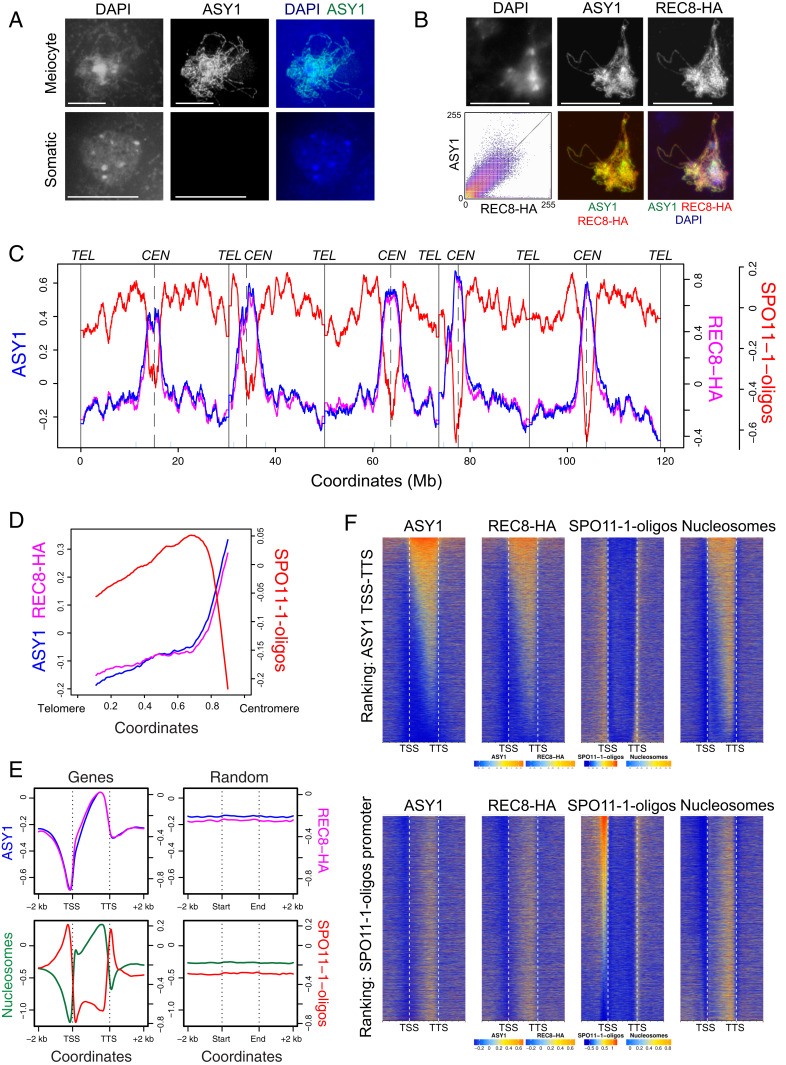
The landscape of ASY1 ChIP-seq enrichment throughout the *Arabidopsis* genome. (*A*) Meiotic cells in early prophase I or adjacent somatic cells immunostained for ASY1 (green) and stained for chromatin (DAPI, blue). (*B*) Male meiocytes in early prophase I immunostained for ASY1 (green) and REC8-HA (red) and stained for chromatin (DAPI, blue). (*Inset*) Correlation plot of ASY1 and REC8-HA signal intensity (*n* = 10 cells). (Scale bars, 10 μM.) (*C*) ASY1 (blue) and REC8-HA (pink) ChIP-seq enrichment [log_2_(ChIP/input)] and SPO11-1-oligos [log_2_(SPO11-1-oligos/gDNA), red] along the *Arabidopsis* genome in adjacent 10-kb windows, smoothed using a moving average (25, 30). Vertical solid and dotted lines indicate the telomeres and centromeres, respectively. The pericentromere boundaries are indicated by pale blue ticks on the *x* axis. (*D*) Data as in *C*, but analyzing proportionally scaled chromosome arms from telomeres to centromeres. (*E*) Data as in *C*, but showing mean coverage profiles for ASY1 (blue), REC8-HA (pink), nucleosomes [log_2_(MNase-seq/gDNA), green], and SPO11-1-oligos (red) over proportionally scaled windows between gene transcriptional start (TSS) and termination (TTS) sites and 2-kb flanking regions. The same number of randomly positioned windows of the same widths were analyzed as a control. (*F*) Data as in *C*, but analyzed as heat maps within genes and 2-kb flanking regions. Genes were ranked by ASY1 levels in gene bodies (TSS–TTS; *Upper*) or by promoter SPO11-1-oligos (*Lower*). Shading is equal to defined quantiles of coverage values mapped linearly to a vector of six colors.

To investigate the genome-wide localization of ASY1, we performed ChIP-seq using a polyclonal rabbit α-ASY1 antibody raised against full-length recombinant protein (12). Immunostaining of anther spreads using the α-ASY1 antibody shows specific detection in meiocytes, and not in adjacent somatic cells (Fig. 1*A*). Coimmunostaining of ASY1 and REC8-HA showed highly correlated signals during early prophase I (signal intensity correlation *r =* 0.76–0.85, *n* = 10; Fig. 1*B*). We performed ChIP-seq using the α-ASY1 antibody on meiotic-stage floral buds and obtained two independent biological replicate libraries with 26,488,565 and 39,593,737 mapping read pairs (17.5× and 28.2× genome coverage, respectively; *SI Appendix*, Table S1). The ChIP-seq replicates are highly correlated at the genome scale (*r*_*s*_ = 0.91 using 10-kb adjacent windows; *SI Appendix*, Table S2). To determine the specificity of ASY1 ChIP-seq enrichment, two controls were performed. First, the α-ASY1 antibody was used for ChIP-seq from leaf tissue, where ASY1 is not expressed (12). Second, preimmune serum was used for ChIP-seq from floral tissue. After deduplication, only 0.29% and 0.39% of reads in these libraries mapped to the *Arabidopsis* genome (*SI Appendix*, Table S1). In contrast, 90.1% and 93.2% of deduplicated ASY1 ChIP-seq reads were mapped (*SI Appendix*, Table S1). This demonstrates the low background of reads that map to the *Arabidopsis* genome obtained from our ChIP protocol, in the absence of the epitope or the α-ASY1 antibody. For further analysis, ASY1 ChIP-seq libraries were normalized using an input chromatin library to generate log_2_(ChIP/input) enrichment values across the genome (Fig. 1*C*).

At the genome scale, we observed highest ASY1 ChIP-seq enrichment over the centromeric and pericentromeric regions (Fig. 1*C* and *SI Appendix*, Fig. S1). An ascending gradient of ASY1 ChIP-seq enrichment was observed from telomeres to centromeres, with the sharpest increase observed as the centromeres are approached (Fig. 1*D*). We observed a striking positive correlation between ASY1 and REC8-HA ChIP-seq enrichment (e.g., *r*_*s*_ = 0.88**–**0.93 at 10-kb scale; Fig. 1*C* and *SI Appendix*, Fig. S1 and Table S2) (30), which is consistent with their highly correlated immunostaining patterns (Fig. 1*B*). We compared ASY1 ChIP-seq enrichment to DSBs using SPO11-1-oligos as a marker (Fig. 1*C*) (25). At the chromosome scale, the regions in proximity to the centromere where ASY1 is highest have the lowest DSBs (Fig. 1*C*). However, when considering the chromosome arms alone, ASY1 and SPO11-1-oligos show a weak positive correlation (*r*_*s*_ = 0.48 at 10-kb scale; Fig. 1 *C* and *D* and *SI Appendix*, Fig. S1). At the fine scale, SPO11-1-oligos are highest within nucleosome-depleted gene promoters and terminators (Fig. 1*E*) (25). In contrast, ASY1 and REC8 are highest within nucleosome-enriched gene bodies (Fig. 1*E*) (30). Variation in ASY1 enrichment within genes correlates positively with REC8 and nucleosome occupancy (MNase-seq), but does not correlate with SPO11-1-oligos in gene promoters or terminators (Fig. 1*F*). Equally, variation between genes in promoter SPO11-1-oligo levels does not correlate with ASY1 or REC8 ChIP-seq enrichment within gene bodies (Fig. 1*F*).

### Telomere-Led Recombination Dominates in *asy1* Mutants.

As we observed a gradient of ASY1 ChIP-seq enrichment between the telomeres and centromeres, we sought to investigate crossover patterning along chromosomes in *asy1* mutants. Homozygous *asy1* mutants have low fertility due to reduced chiasmata and a high incidence of univalent chromosomes at metaphase I, which leads to aneuploid gametes (9, 15). Despite this, low numbers of viable progeny can be obtained from *asy1* homozygotes. Therefore, we crossed *asy1/+* individuals in Col [*asy1-4* (15)] and Ws-4 [hereafter Ws; *asy1-3/+* (32)] backgrounds to generate wild type or *asy1* Col×Ws F_1_ plants. The F_1_ plants were self-fertilized, and 187 wild type and 169 *asy1* F_2_ progeny were generated and used for DNA sequencing. The TIGER pipeline was used to identify crossover locations from the sequencing data (*SI Appendix*, Table S3) (33).

**Fig. 2.**
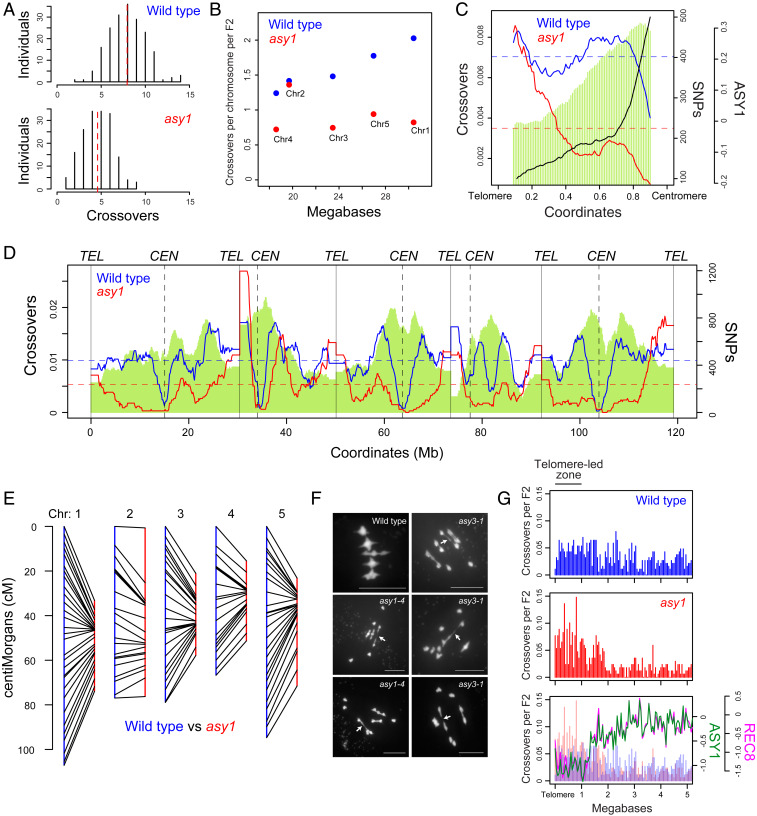
Telomere-led recombination predominates in *asy1*. (*A*) Histograms of crossovers per F_2_ individual for wild type and *asy1*. Red dashed lines indicate mean values. (*B*) crossovers per chromosome per F_2_ for wild type (blue) and *asy1* (red) plotted against chromosome length in megabases. (*C*) crossover frequency in wild type (blue) and *asy1* (red), and ASY1 ChIP-seq enrichment [log_2_(ChIP/input), black] analyzed along proportionally scaled chromosome arms, oriented from telomeres to centromeres. Col×Ws SNP density is shown by green shading. (*D*) Crossover frequency (crossovers/150 kb per F_2_) plotted along the *Arabidopsis* genome for wild type (blue) and *asy1* (red), with Col×Ws SNP density (green) shaded. Telomere and centromere positions are indicated by vertical solid and dotted lines, respectively. (*E*) Comparison of F_2_ genetic map lengths (in centimorgans, cM) in wild type (blue) and *asy1* (red). (*F*) Spreads of male meiocytes at metaphase I in wild type (Col) and in *asy1* and *asy3* mutants with DAPI-stained chromatin. Arrows indicate potentially distal chiasmata locations. (Scale bars, 10 μM.) (*G*) Crossover positions analyzed relative to the closest telomere in wild type (blue) and *asy1* (red). The lower plot shows ASY1 (green) and REC8-HA (pink) ChIP-seq enrichment [log_2_(ChIP/input)] analyzed over the same regions.

As expected, a significant decrease in crossovers per F_2_ was observed in *asy1* (mean = 4.6) compared to wild type [mean = 7.9; Mann–Whitney–Wilcoxon (MWW) test *P* = 4.37 × 10^−37^; Fig. 2*A* and *SI Appendix*, Table S3]. However, the number of crossovers observed per *asy1* F_2_ individual was higher than predicted from bivalent counts per meiosis in *asy1-3* [mean = 1.5 (32)] and *asy1-4* [mean = 1.9 (9); Fig. 2*A* and *SI Appendix*, Table S3]. This may reflect generation of viable F_2_ plants selecting for gametes with at least one crossover per chromosome in order to balance segregation at metaphase I. Alternatively, as chiasmata measurements are made from male meiosis, whereas F_2_ crossover data reflect both male and female meiosis, this could indicate sex differences in crossover reduction in *asy1*. A further possibility is that closely spaced crossovers may be counted as single chiasmata in *asy1*, causing an underestimation of recombination. In wild type, crossover number per chromosome is positively correlated with physical length (*r* = 0.98, *P =* 3.4 × 10^−3^), whereas no significant correlation exists in *asy1* (Fig. 2*B*). Exceptionally, chromosome 2 shows a crossover frequency close to wild type in *asy1*, with a striking increase on the short, nucleolar organizing region (NOR)-bearing arm (Fig. 2 *B* and *D* and *SI Appendix*, Table S3). This is consistent with chiasmata and fluorescent in situ hybridization (FISH) analysis in *asy1* mutants in the Ws accession, where the short arm of chromosome 2 also showed high chiasmata frequency (34). Interestingly, in Col×Ws F_1_ hybrids, *NOR2* rDNA gene clusters are transcriptionally silenced, whereas *NOR4* on chromosome 4 are expressed (35). Nucleolar silencing is known to involve formation of heterochromatin at the transcriptionally repressed *NOR* (36). Hence, heterochromatin formation at *NOR2* could contribute to closer alignment of homologs and thereby promote crossover formation on chromosome 2 in *asy1*.

When recombination was analyzed along scaled telomere–centromere axes, we observed a strong bias of *asy1* crossover formation toward the subtelomeric regions (Fig. 2 *C*–*E*). Analysis of chiasmata in *asy1*, *asy3*, and *asy4* axis mutants has shown a high incidence of rod bivalent configurations (Fig. 2*F*) (9, 10, 34), which may reflect distal crossover locations. To investigate recombination in relation to telomere position, we assigned each crossover a distance to its nearest telomere and plotted events on a common axis (Fig. 2*G* and *SI Appendix*, Fig. S2). The crossover counts observed were analyzed in windows relative to the telomere in wild type and *asy1* and used to perform χ^2^ tests, with correction for multiple testing (*SI Appendix*, Table S4). We observed that windows in the first megabase of chromosomes show significantly greater crossovers in *asy1* (Fig. 2*G* and *SI Appendix*, Figs. S2 and S3 and Table S4), which we term the telomere-led zone (TLZ). Interestingly, the TLZ corresponds to distal regions that have relatively low ASY1 and REC8 ChIP-seq enrichment in wild type (Fig. 2*G*). At the fine scale, we observed that *asy1* crossovers show a preference to form in nucleosome-depleted, AT-rich regions with higher-than-average SPO11-1-oligos, which were similar to wild type crossovers (*SI Appendix*, Fig. S4) (25). Hence, although crossovers are highly distalized in *asy1* mutants, they retain a local bias toward chromatin and sequence features related to elevated DSB levels (25).

### Crossovers Remodel toward Telomeric Regions in *asy1/+* Heterozygotes.

**Fig. 3.**
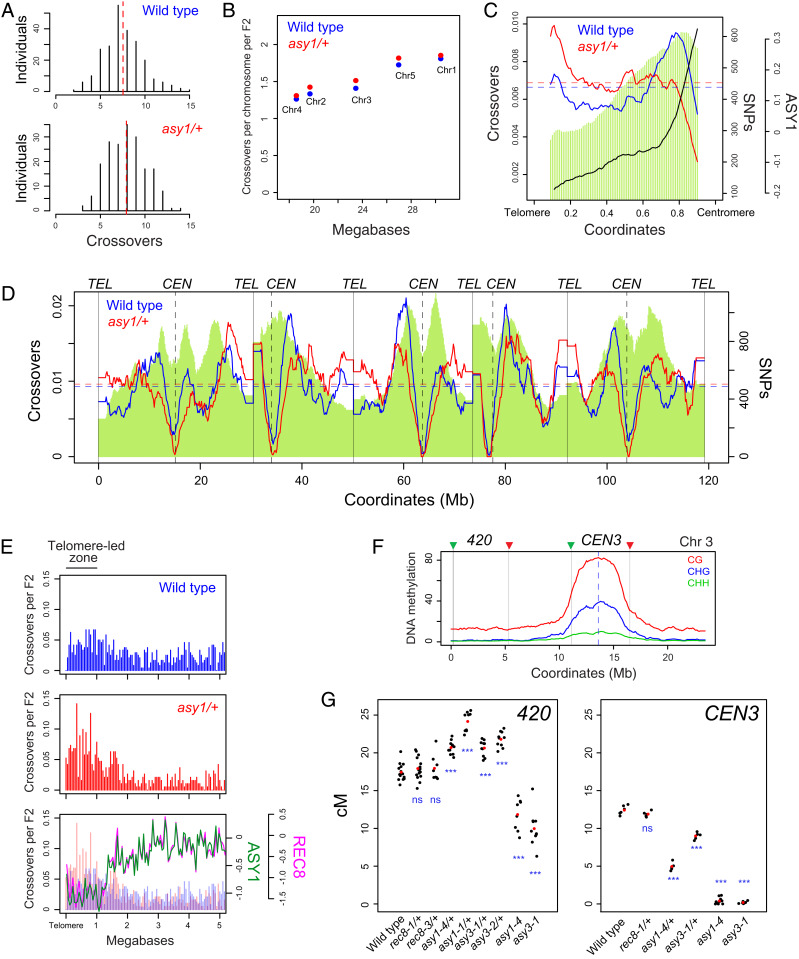
Distal increases in crossover frequency in *asy1/+* and *asy3/+* axis heterozygotes. (*A*) Histograms of crossovers per F_2_ individual for wild type and *asy1/+*. Red dashed lines indicate mean values. (*B*) Crossovers per chromosome per F_2_ for wild type (blue) and *asy1/+* (red) plotted against chromosome length in megabases. (*C*) Crossover frequency in wild type (blue) and *asy1/+* (red) and ASY1 ChIP-seq enrichment [log_2_(ChIP/input), black] analyzed along proportionally scaled chromosome arms, oriented from telomeres to centromeres. Col×Ler SNP density is shown by green shading. (*D*) Crossover frequency (crossovers/150 kb per F_2_) plotted along the *Arabidopsis* genome for wild type (blue) and *asy1/+* (red), with Col×Ler SNP density (green) shaded. Telomere and centromere positions are indicated by vertical solid and dotted lines, respectively. (*E*) Crossovers analyzed relative to the closest telomere in wild type (blue) and *asy1/+* (red). The lower plot shows ASY1 (green) and REC8-HA (pink) ChIP-seq enrichment [log_2_(ChIP/input)] analyzed over the same regions. (*F*) DNA methylation (CG, CHG, CHH) in wild type (Col) is plotted along chromosome 3, and the positions of the *420* and *CEN3* FTL intervals are indicated. (*G*) Crossover frequency (in cM) within the *420* and *CEN3* FTL intervals in the indicated genotypes. Black dots represent replicate measurements, and red dots represent mean values. To assess significant differences, *t* tests were performed (n.s., not significant; ***P < 0.01).

We next sought to investigate whether *asy1/+* heterozygotes associate with remodeled crossover frequency. We self-fertilized *asy1-4*/+ Col×Ler F_1_ hybrids to generate 191 F_2_ plants, which were then sequenced (*SI Appendix*, Table S3). These data were compared to crossovers previously mapped in a control Col×Ler wild type F_2_ population (37). Crossovers per F_2_ were not significantly different between wild type (mean = 7.54) and *asy1-4/+* (mean = 7.92) populations (MWW test, *P* = 0.059; Fig. 3*A* and *SI Appendix*, Table S3). A positive correlation exists between number of crossovers per chromosome and physical chromosome length in both *asy1/+* (*r =* 0.97 *P* = 6.17 × 10^−3^) and wild type (*r =* 0.97 *P* = 4.83 × 10^−3^) Col×Ler (Fig. 3*B*). Hence, global crossover numbers are maintained in *asy1*/+ heterozygotes, relative to wild type.

At the chromosome scale, despite crossover numbers being maintained, we observed that the *asy1/+* recombination landscape was remodeled (Fig. 3 *C* and *D*). Specifically, crossovers increased in the distal subtelomeric regions in *asy1/+* compared to wild type at the expense of the pericentromeric regions (Fig. 3 *C* and *D*). The centromeres remained crossover–suppressed in both wild type and *asy1*/+ populations (Fig. 3 *C* and *D*). We repeated analysis of crossover positions relative to the nearest telomere and compared crossover counts between wild type and *asy1/+* using χ^2^ tests (Fig. 3*E* and *SI Appendix*, Table S4). This identified the first megabase within the subtelomeric regions as showing significantly elevated crossovers in *asy1/+* compared to wild type (Fig. 3*E* and *SI Appendix*, Fig. S3 and Table S4), which overlaps with the TLZ observed in *asy1* (Fig. 2*G*). As noted, the TLZ contains regions of relatively low ASY1 and REC8 ChIP-seq enrichment in wild type, which may explain the sensitivity of crossovers in these regions to reduced *ASY1* gene dosage (Fig. 3*E*). These findings demonstrate that *asy1/+* heterozygotes maintain crossover numbers, but show remodeling of recombination toward distal regions.

### Crossovers Are Sensitive to *ASY1* and *ASY3* Gene Dosage, but Not *REC8*.

To further investigate changes to crossover frequency associated with meiotic axis gene dosage, we used fluorescent tagged lines (FTLs) (38, 39). FTL intervals are defined by T-DNA insertions that express different colors of fluorescent protein (green, red, or blue) in pollen (*LAT52* promoter) or seed (*NapA* promoter) (38, 39). When an individual is hemizygous for linked T-DNAs, patterns of fluorescence in pollen or seed can be used to quantify crossover frequency within the intervals flanked by the T-DNAs (40, 41). We crossed the subtelomeric FTL interval *420* on chromosome 3 to *asy1/+*, *asy3/+*, and *rec8/+*, using two independent alleles in each case (Fig. 3*F* and *SI Appendix*, Table S5). We observed that all *asy1/+* and *asy3/+* heterozygotes showed significantly increased *420* crossover frequency compared to wild type (*t* test *P* value range = 2.39 × 10^−7^ to 2.00 × 10^−9^; Fig. 3*G* and *SI Appendix*, Table S5). In contrast, *rec8/+* heterozygotes showed no significant difference (*t* test *P* = 0.26; Fig. 3*G* and *SI Appendix*, Table S5). As *420* is located distally, we observed a relatively high genetic distance in *asy1* and *asy3* (∼10 cM; Fig. 3*F* and *SI Appendix*, Table S5) despite these backgrounds having reduced crossovers genome-wide (Fig. 2) (9, 11). It is not possible to measure FTLs in *rec8* homozygotes, as they are completely sterile (7, 42, 43).

Due to the remodeling of crossovers along the chromosomes observed in *asy1/+* (Fig. 3*C*), we also measured recombination using the *CEN3* FTL, which spans the DNA methylated centromere and pericentromere of chromosome 3 (Fig. 3*F* and *SI Appendix*, Table S6). *CEN3* showed a significant decrease in crossover frequency in *asy1/+* and *asy3/+* heterozygotes compared to wild type (*t* test *P* = 4.60 × 10^−9^ and 2.05 × 10^−6^; Fig. 3*G* and *SI Appendix*, Table S6). No significant difference in *CEN3* crossover frequency was observed between *rec8/+* and wild type (*t* test *P* = 0.11; Fig. 3*G* and *SI Appendix*, Table S6). In *asy1* and *asy3* homozygotes, *CEN3* crossovers were virtually eliminated compared to wild type (*t* test *P* = 5.85 × 10^−11^ and 1.40 × 10^−8^; Fig. 3*G* and *SI Appendix*, Table S6), consistent with telomere-led recombination dominating in these backgrounds. These experiments demonstrate remodeling of the crossover landscape toward the telomeres via reduced gene dosage of *ASY1* and *ASY3*, but not *REC8*.

### Cytogenetic Analysis of Meiosis in *asy1/+* and *asy3/+* Heterozygotes.

**Fig. 4.**
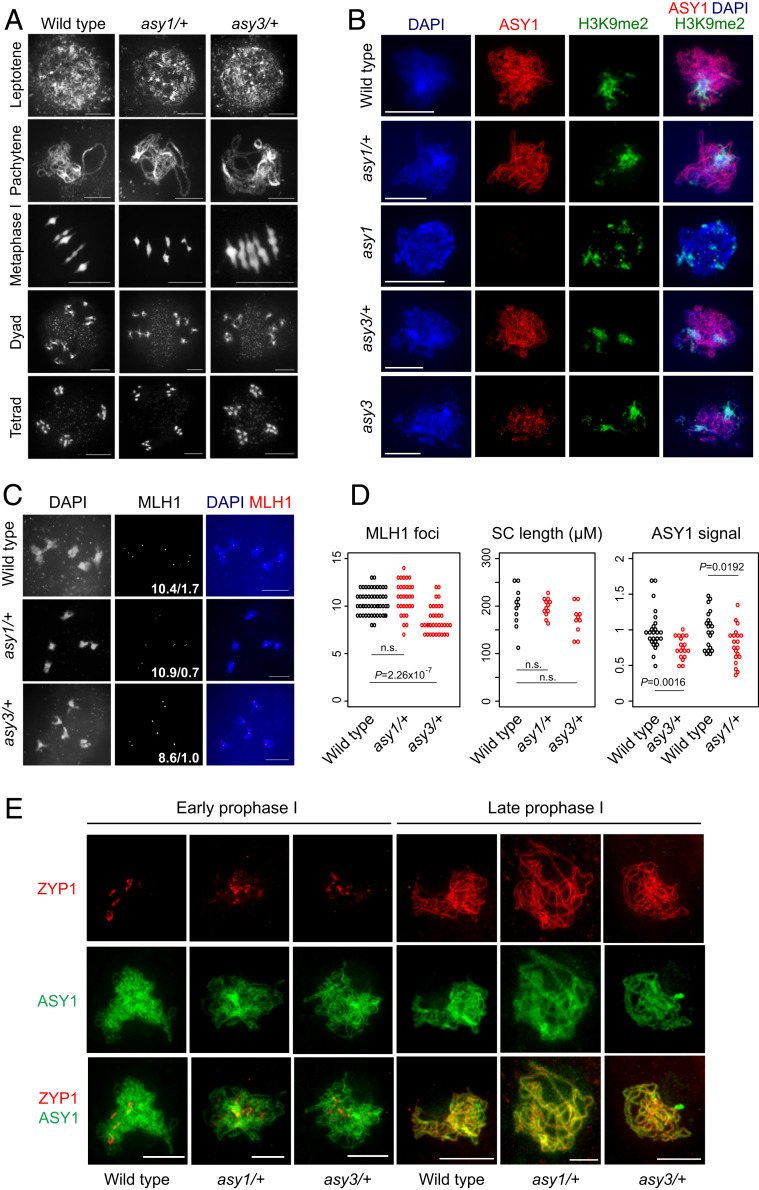
Cytological analysis of meiosis in *asy1/+* and *asy3/+* heterozygotes. (*A*) Spreads of wild type, *asy1-4/+*, and *asy3-1/+* male meiocytes at the labeled stages of meiosis, with chromatin stained by DAPI. (*B*) Male meiocytes immunostained for ASY1 (red) and H3K9me2 (green) and stained for DNA (DAPI, blue) in the indicated genotypes. (*C*) Pollen mother cells immunostained for MLH1 (red) at diakinesis stage in wild type, *asy1-4/+*, and *asy3-1/+*. Chromatin was stained with DAPI (blue). The mean number of MLH1 foci and the subset of foci overlapping heterochromatin are printed for each genotype. (*D*) Quantification of MLH1 foci per meiocyte, ASY1 immunostaining signal, and ZYP1 immunostaining-derived synaptonemal complex (SC) length (in μM) per cell in the indicated genotypes. Statistical significance was assessed using MWW tests. (*E*) Male meiocytes immunostained for ASY1 (green) and ZYP1 (red) at early and late prophase I in wild type, *asy1-4/+*, and *asy3-1/+*. (Scale bars, 10 μM.)

We next analyzed meiotic progression of *asy1/+* and *asy3/+* heterozygotes using chromosome spreads of pollen mother cells and DAPI staining of chromatin (Fig. 4*A*). Chromosomes paired normally at pachytene in the *asy1/+* and *asy3/+* heterozygotes, and heterochromatic regions of dense DAPI staining were visible during prophase I (Fig. 4*A*). Five bivalents were detected at diakinesis, and no missegregation of chromosomes was observed at anaphase I or meiosis II in *asy1/+* and *asy3/+* (Fig. 4*A*). Consistently, no significant decrease in *asy1/+* or *asy3/+* seed count or pollen viability was observed (*SI Appendix*, Tables S7 and S8). To further assess chromatin organization, we immunostained male meiocytes for ASY1 and the heterochromatic histone modification H3K9me2 in wild type, *asy1/+*, *asy1*, *asy3/+*, and *asy3* (Fig. 4*B*) (26, 44). H3K9me2 staining on chromosomes was observed in all genotypes, consistent with normal heterochromatin formation (Fig. 4*B*). ASY1 was undetectable in *asy1*, and showed an altered punctate staining pattern in *asy3*, as reported (Fig. 4*B*) (9).

To cytologically analyze Class I crossovers, we immunostained diakinesis-stage male meiocytes using α-MLH1 antibodies and stained chromatin with DAPI (Fig. 4*C* and *SI Appendix*, Fig. S5 and Table S9). We did not observe significant differences in total MLH1 foci between *asy1/+* and wild type (MWW test *P* = 0.128), but a small yet significant decrease occurred in *asy3/+* (MWW test *P =* 2.26 × 10^−7^; Fig. 4 *C* and *D* and *SI Appendix*, Table S9). We quantified MLH1 foci overlapping pericentromeric heterochromatin, defined by DAPI-dense regions, and observed a significant decrease in *asy1/+* and *asy3/+* compared to wild type (MWW test *P* = 4.53 × 10^−5^ and *P* = 8.94 × 10^−4^; Fig. 4 *C* and *D* and *SI Appendix*, Fig. S5 and Table S9). This is further consistent with distalization of crossovers away from centromere-proximal regions in *asy1/+* and *asy3/+* heterozygotes.

To investigate the effect of *asy1/+* heterozygosity on axis loading of ASY1, we quantified α-ASY1 immunostained signal intensity during early prophase I and observed a 21% reduction in *asy1/+* compared to wild type (MWW test *P* = 0.019; Fig. 4 *D* and *E* and *SI Appendix*, Table S10). As ASY3 is required for polymerization of ASY1 during meiosis (Fig. 4*B*) (9), we also quantified α-ASY1 signal intensity in *asy3/+* and observed a 25% reduction compared to wild type (MWW test *P* = 1.6 × 10^−3^; Fig. 4 *D* and *E* and *SI Appendix*, Table S10). We immunostained pachytene-stage cells for the synaptonemal complex (SC) transverse filament ZYP1 (45). Continuous ZYP1 signal was observed along chromosomes in *asy1/+* and *asy3/+* compared to wild type, and no significant differences in SC length were observed (MWW tests *P* = 0.74 and *P* = 0.27; Fig. 4 *D* and *E* and *SI Appendix*, Table S11). Hence, although we detect a reduction in ASY1 loading in *asy1/+* and *asy3/+* heterozygotes, full pairing and synapsis occurs in these backgrounds.

### Crossover Interference Is Maintained in *asy1/+* but Is Absent in *asy1*.

We next investigated crossover interference in *asy1/+* and *asy1* using analysis of double crossover (DCO) events. As we sequenced F_2_ individuals, which derive from two independent meioses, in some cases, there is uncertainty about whether an observed DCO occurs in *cis* or *trans* (*SI Appendix*, Fig. S6) (46). Importantly, only *cis* DCOs, which occurred in the same meiosis, are relevant for measurement of crossover interference (46). In our F_2_ data, a subset of *cis* DCOs can be identified from Col–Het–Col, Ler–Het–Ler, or Ws–Het–Ws genotype blocks (*SI Appendix*, Fig. S6) (46, 47). Therefore, we filtered for DCOs following this genotype pattern, which resulted in 118, 86, 98, and 73 DCOs in the Col×Ler wild type and *asy1/+* and Col×Ws wild type and *asy1* populations, respectively (*SI Appendix*, Table S12). For each population, a matched set of randomly positioned DCOs of the same widths was generated as a control comparison. We analyzed *recq4a recq4b* crossover data in the same way (37), where the interference-insensitive Class II crossover repair pathway is greatly increased (37, 48). In *asy1*, the majority of the remaining crossovers have been shown to be dependent on the Class I pathway (9, 15).

**Fig. 5.**
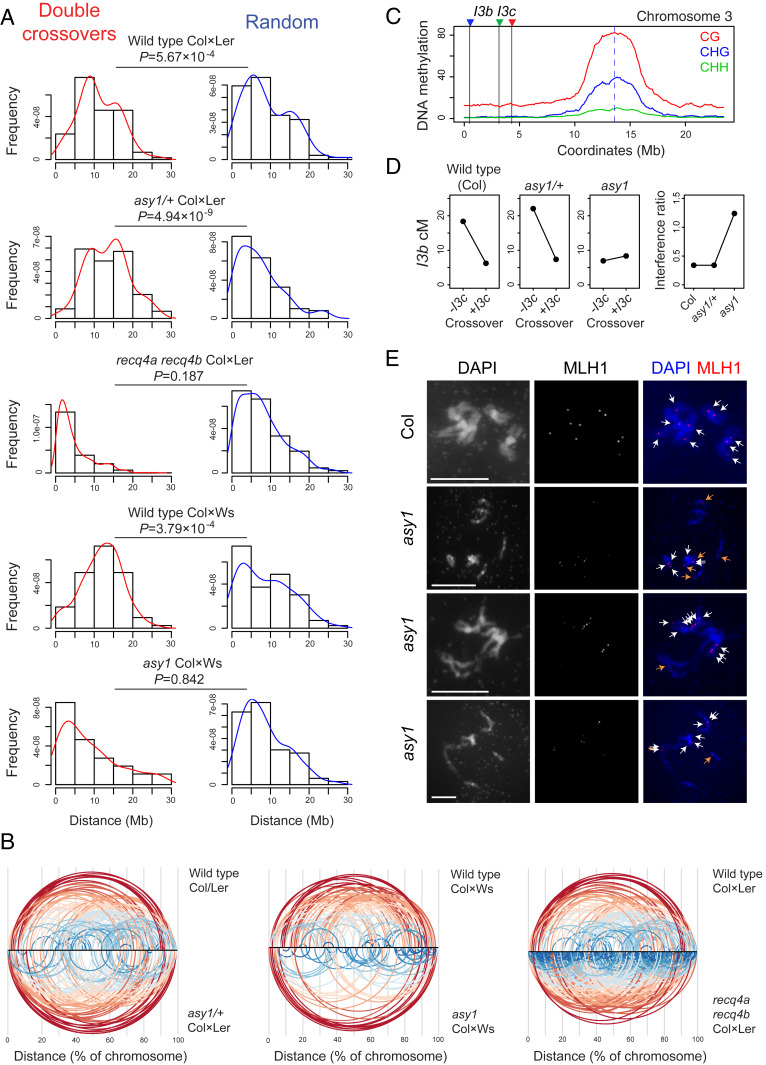
Crossover interference is maintained in *asy1/+* but is absent in *asy1*. (*A*) Histograms showing the distribution of observed double crossover distances (DCOs, red) in megabases in wild type, *recq4a recq4b* (37), and *asy1/+* Col×Ler F_2_ individuals or wild type and *asy1* Col×Ws F_2_ individuals. Alongside are identical histograms showing the distribution of matched randomly generated distances (blue). Mann–Whitney–Wilcoxon (MWW) tests were performed to assess significant differences between observed DCOs and random, with *P* values indicated. (*B*) Diagrams showing spacing of identified DCOs along the proportional physical length of chromosomes (as percentages). DCOs are connected via arcs and color-coded proportional to the distance between them (red, greatest; blue, smallest). (*C*) DNA methylation (CG, CHG, CHH) in wild type plotted along chromosome 3 with the positions of the *I3bc* FTL T-DNAs indicated by vertical lines and colored triangles. (*D*) Crossover frequency (in cM) within *I3b*, contingent on crossover in the adjacent interval *I3c*, in wild type, *asy1/+*, and *asy1*. Interference ratios calculated from the *I3bc* data are plotted for the same genotypes. (*E*) Representative images of pollen mother cells immunostained for MLH1 (red) at diakinesis stage in wild type and *asy1.* Chromatin was stained with DAPI (blue). White and orange arrows in the merged images indicate MLH1 foci located on bivalents or univalents, respectively. (Scale bars, 10 μM.)

Consistent with the action of crossover interference, distances between observed DCOs were significantly greater than random in both wild type Col×Ler (MWW *P* = 5.67 × 10^−4^) and Col×Ws (MWW test *P* = 3.79 × 10^−4^; Fig. 5 *A* and *B* and *SI Appendix*, Table S12). The *asy1/+* DCOs were also more widely spaced than random (MWW *P* = 4.94 × 10^−9^), which is consistent with normal crossover interference in this background (Fig. 5 *A* and *B* and *SI Appendix*, Table S12). In contrast, the spacing of DCOs in *asy1* was not significantly different from random (MWW *P* = 0.842; Fig. 5 *A* and *B* and *SI Appendix*, Table S12), showing an absence of detectable crossover interference. Using the same analysis method, DCOs observed in *recq4a recq4b* were not significantly different from random (*P* = 0.187), as expected due to greatly elevated noninterfering crossover repair (37, 48).

To independently measure crossover interference in wild type, *asy1/+*, and *asy1*, we used the distally located three-color FTL interval *I3bc*, which overlaps the *420* FTL interval on chromosome 3 (Fig. 5*C*, Table 1, and *SI Appendix*, Fig. S7). We measured crossover frequency between the *I3bc* T-DNAs in a *qrt1* background, where the four sister haploid cells produced from each male meiosis remain physically attached, allowing tetrad pollen analysis (Table 1) (38). To estimate crossover interference, we calculated *I3b* crossover frequency with and without a crossover in the adjacent *I3c* interval (Table 1). These measurements are used to calculate an interference ratio, where values closer to 0 indicate stronger interference and values close to 1 indicate an absence of interference (38).

**Table 1.** Pollen tetrad analysis of crossover frequency and interference within the *I3bc* FTL intervals in wild type (Col), *asy1-4/+*, and *asy1-4*

Tetrad class	Wild type (Col)	*asy1-4/+*	*asy1-4*
A – NCO	2,338	2,092	913
B – SCO *I3c*	328	317	46
C – SCO *I3b*	1,313	1,574	113
D – SCO *I3b* and SCO *I3c*	12	16	6
E – SCO *I3b* and SCO *I3c*	8	17	1
F – SCO *I3b* and SCO *I3c*	13	10	0
G – SCO *I3b* and SCO *I3c*	14	13	1
H – DCO *I3c*	3	2	2
I – DCO *I3b*	5	8	5
J – DCO *I3c* and SCO *I3b*	0	0	1
K – SCO *I3c* and DCO *I3b*	0	0	1
L – DCO *I3c* and DCO *I3b*	0	0	0
Total	4,034	4,049	1,089
Genetic distance			
* I3b* cM (*P* value)	17.23	20.72 (5.20 × 10^−9^)	7.02 (3.95 × 10^−49^)
* I3c* cM (*P* value)	4.87	4.75 (0.895)	3.35 (1.06 × 10^−5^)
* I3b* cM without adjacent *I3c* CO	18.37	22.07	6.94
* I3b* cM with adjacent *I3c* CO	6.22	7.47	8.26
Interference ratio	0.34	0.34	1.24
* P* value	—	0.997	7.20 × 10^−6^

Fluorescent pollen tetrads were classified into one of 12 color patterns: non–crossover (A – NCO), single *I3c* crossover (B – SCO *I3c*), single *I3b* crossover (C – SCO *I3b*), two-strand *I3bc* double crossovers (D – SCO *I3b* and SCO *I3c*), three-strand double crossovers type 1 (E – SCO *I3b* and SCO *I3c*), three-strand double crossovers type 2 (F – SCO *I3b* and SCO *I3c*), four-strand double crossovers (G – SCO *I3b* and SCO *I3c*), four-strand double crossovers within interval *I3c* (H – DCO *I3c*), four-strand double crossovers within interval *I3b* (I – DCO *I3b*), double *I3c* crossovers and single *I3b* crossover (J – DCO *I3c* and SCO *I3b*), single *I3c* crossover and double *I3b* crossovers (K – SCO *I3c* and DCO *I3b*), and double *I3c* crossovers and double *I3b* crossovers (L – DCO *I3c* and DCO *I3b*). Recombination frequency was measured using the Perkins method (38). Interference ratios and statistical tests were calculated using the Malkova method as described (38). The locations of the *I3bc* FTL T-DNAs are 498,916 (CFP), 3,126,994 (YFP), and 4,319,513 (dsRed2) bp on chromosome 3 (40).

In wild type, the more distal interval *I3b* shows higher crossover frequency than *I3c* and an interference ratio of 0.34 (Fig. 5*D* and Table 1). In *asy1/+*, a significant increase in *I3b* crossover frequency occurred compared to wild type (Perkins *P* = 5.20 × 10^−9^), whereas *I3c* was not significantly changed (Fig. 5*D* and Table 1). Consistent with our previous observations, no significant difference was observed in the interference ratio between wild type and *asy1/+* (Perkins *P* = 0.997; Fig. 5*D* and Table 1). In *asy1*, both genetic intervals showed a significant reduction in crossover frequency compared to wild type (*I3b* Perkins *P* = 3.95 × 10^−49^ and *I3c* Perkins *P* = 1.06 × 10^−5^), although the more distal *I3b* interval maintains a higher level of crossovers than *I3c* (Fig. 5*D* and Table 1). In contrast to *asy1/+*, the *asy1* homozygotes showed an interference ratio of 1.24 that was significantly different than wild type (Perkins *P* = 7.20 × 10^−6^; Fig. 5*D* and Table 1), further consistent with an absence of crossover interference.

Finally, to investigate Class I crossovers in wild type and *asy1*, we immunostained MLH1 at diakinesis stage and stained DNA with DAPI (Fig. 5*E* and *SI Appendix*, Tables S9 and S13). In wild type (Col), no univalent chromosomes are observed, and, on average, 10.4 MLH1 foci occurred on the bivalents (Fig. 5*E* and *SI Appendix*, Tables S9 and S13). In *asy1*, we observed a higher incidence of univalent chromosomes per cell (mean = 5.6) compared to bivalents (mean = 2.6; Fig. 5*E* and *SI Appendix*, Table S13). Interestingly, we observed MLH1 foci on both univalents (mean = 5.3) and bivalents (mean = 5) in *asy1* (Fig. 5*E* and *SI Appendix*, Table S13). The presence of MLH1 foci on *asy1* bivalents is consistent with crossover formation via the Class I pathway. MLH1 foci have been reported on univalent chromosomes in *dmc1* and in haploid meiosis and may represent sites of intersister repair (49). In order to estimate crossover interference, we measured MLH1 interfoci distances on bivalents in wild type and *asy1* (*SI Appendix*, Table S14). We observed that MLH1 foci were significantly closer in *asy1* compared to wild type (MWW test *P* = 2.19 × 10^−6^; *SI Appendix*, Table S14), which is further consistent with a loss of crossover interference. Therefore, our combined analysis of DCO spacing from sequencing data, fluorescent pollen tetrads, and MLH1 foci show that crossover interference is absent in *asy1*.

## Discussion

**Fig. 6.**
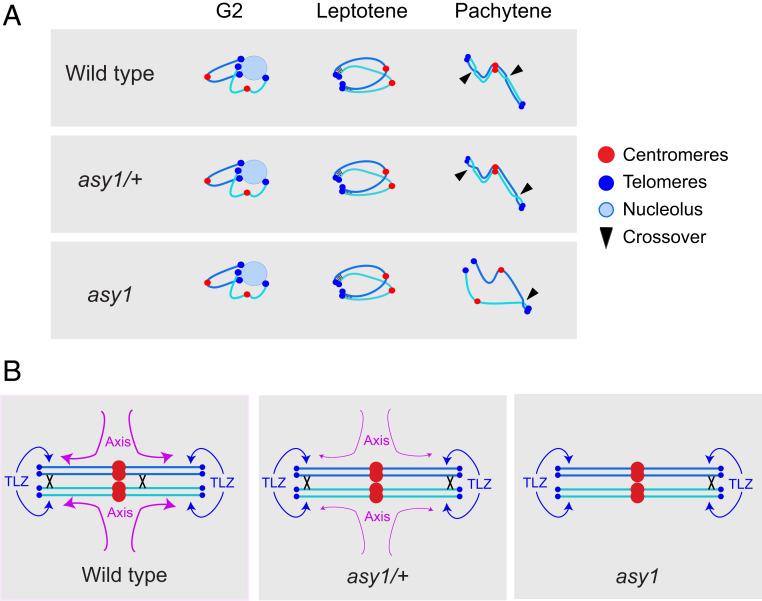
ASY1 acts as a gene dosage-sensitive antagonist of telomere-led recombination and mediates crossover interference. (*A*) A single pair of homologs is represented at the G2, leptotene, and pachytene stages of meiosis in wild type, *asy1/+*, and *asy1*. Centromeres are represented as red dots, telomeres as blue dots; the nucleolus is a pale blue circle, and crossover positions are indicated by black triangles. Telomere-led alignment of homologs is shown with black bars connecting two chromosomes at leptotene stage. (*B*) A single aligned pair of homologs is shown, with the positions of crossovers indicated by crosses in wild type, *asy1/+*, and *asy1*. Crossovers are promoted in proximity to the telomere-led zone (TLZ, blue). ASY1 (purple) loading antagonizes the TLZ and promotes interstitial and proximal crossovers via interference. Note that this model represents metacentric chromosomes such as chromosomes 1, 3, and 5. The acrocentric chromosomes 2 and 4, which bear nucleolar organizing regions (NORs) on their short arms, may differ in crossover patterning.

Our data inform a model for how ASY1 and the meiotic axis pattern crossover frequency along plant chromosomes (Fig. 6). Previous work has shown that *asy1* mutants undergo normal telomere clustering, formation of meiotic DSB foci during early leptotene, and polymerization of an axial structure marked by REC8 and ASY3 (Fig. 6*A*) (9, 15, 31). However, DMC1 foci dynamics are altered in *asy1*, resulting in a failure of interhomolog recombination and depletion of crossovers (9, 15). Using high-resolution mapping of crossovers via sequencing F_2_ plants, we show that recombination becomes largely restricted to a telomere-led zone (TLZ) in *asy1* homozygotes (Fig. 6*B*). We propose that the proximity of telomeres during early prophase in *asy1* is responsible for telomere-led recombination (Fig. 6*A*) (31). Telomere-led recombination is active in wild type, but ASY1 antagonizes this activity to promote crossover formation in interstitial and centromere-proximal chromosome regions (Fig. 6*B*). Using ChIP-seq, we observe a gradient of ASY1 enrichment from the telomeres to the centromeres, which is paralleled by REC8 cohesin enrichment (30). We propose that differential ASY1 enrichment represents a mechanism to distribute recombination more evenly along the chromosome arms. However, as heterochromatin increases in proximity to the centromere, this causes suppression of meiotic DSBs and crossovers, despite high levels of ASY1 and REC8 (25, 26, 50).

We show that plants heterozygous for *asy1/+* and *asy3/+* mutations undergo remodeling of the crossover landscape, with a shift toward the distal subtelomeres, at the expense of interstitial and pericentromeric regions. Interestingly, the distal regions that undergo crossover increases in *asy1/+* overlap the TLZ observed in *asy1* and have relatively low levels of ASY1 and REC8 ChIP-seq enrichment in wild type. Using meiotic immunocytology, we quantified a ∼21% reduction in ASY1 loading on chromatin in *asy1/+*. This could indicate a threshold effect over which ASY1 antagonizes telomere-led recombination and promotes crossovers in the chromosome arms, toward the centromere. In *asy1/+* heterozygotes, the distal regions would drop below this putative threshold and the strength of telomere-led recombination would increase. As interference remains operational in *asy1/+*, this would lead to a relative loss of crossovers in the interstitial and pericentromeric regions (Fig. 6*B*). Alternatively, this may reflect a nonlinear effect of decreased ASY1 expression on recombination along the chromosomes. It is notable that genetic variation in axis components, including *ASY1* and *ASY3*, has been strongly associated with adaptation to tetraploidy in *Arabidopsis arenosa*, which may include distalization of crossovers (51, 52). Our results show that gene dosage of *ASY1* and *ASY3* may contribute to these effects, in addition to the influence of specific variants on protein function (51, 52).

Crossover interference is mediated by topoisomerase II and the axis protein Red1 in budding yeast (53), while the SC component SYP-1 has been implicated in *Caenorhabditis elegans* (54, 55). Using analysis of double crossover distances, MLH1 foci, and fluorescent pollen tetrads, we show that ASY1 is required for detectable crossover interference in *Arabidopsis*. Interestingly, crossovers in axis mutants are largely dependent on the Class I interfering repair pathway (9, 15). For example, chiasma are eliminated in *asy3 msh4* double mutants, and we show that MLH1 foci form on *asy1* bivalents (9, 15). Therefore, despite the Class I pathway mediating the majority of crossover formation in *asy1*, interference signaling between recombination sites is inactive. Crossover interference has been proposed to occur via mechanical stress acting across paired homologous chromosomes, which is transmitted along the axis and relieved at crossover designated sites (13, 53). In this respect, ASY1 may mediate crossover interference via transmission of mechanical stress, when chromatin loops connected to the axis undergo cycles of expansion and contraction during early prophase I (13). In the absence of ASY1, the mechanical properties of the axis may be altered, meaning force can no longer be transmitted and crossover interference is not detected. Alternatively, ASY1 may control sensitivity of interhomolog repair sites to the interference signal or mediate transmission of a biochemical signal along the chromosome axis (56). Our work indicates that axis HORMA domain proteins can play a critical role in mediating crossover interference along chromosomes during meiosis.

## Materials and Methods

### Plant Materials.

*Arabidopsis* plants were grown under long day conditions (16 h light/8 h dark) at 20 °C. The following mutant alleles in the Col-0 background were used: *rec8-1* (Salk_091193) (9), *rec8-3* (SAIL_807_B08) (49), *asy1-1* (Salk_144182) (15), *asy1-4* (Salk_046272) (23), *asy3-1* (Salk_143676) (9), and *asy3-2* (SAIL_423_H01) (9). The *asy1-3* allele is in the Ws-4 background (32). The *REC8-HA rec8* line was as reported (30).

### ASY1 Chromatin Immunoprecipitation and Sequencing.

ASY1 ChIP was performed using 8 g of floral buds or leaf tissue. Nuclei isolation and chromatin recovery were performed as described (25, 57). Chromatin was sheared using a Bioruptor instrument (Diagenode) for 15 min at high power alternating 30 s on and 30 s off, followed by 15 min at high power alternating 30 s on and 1 min off. Chromatin immunoprecipitation was performed using an α-ASY1 antibody (12), or the preimmune serum, at a dilution of 1/160. DNA purification, DNA library preparation, and sequencing were performed as described (25).

### ChIP-Seq Data Analysis.

Deduplicated paired-end ASY1, REC8-HA, H3K9me2, H3K4me1, H3K4me2, H3K4me3, H3K27me1, and H3K27me3 ChIP-seq reads, paired-end MNase-seq reads, and single-end SPO11-1-oligo, H2A.Z, and H2A.W reads (25, 30, 58, 59) were aligned to the TAIR10 reference genome using Bowtie2 (version 2.2.9) (60) with the following settings: –very sensitive -p 4 -k 10. For paired-end reads, the Bowtie2 options –no-discordant and –no-mixed were also applied. Prior to alignment, single-end SPO11-1-oligo reads were processed as described (25). Up to 10 valid alignments were reported for each read or read pair. Aligned reads with more than two mismatches were discarded using the SAM optional field “XM:i.” Uniquely aligning reads were extracted by removing alignments with the SAM optional field “XS:i” and with Bowtie2-assigned MAPQ scores lower than 42. Alignments consisting of reads that mapped to multiple loci were filtered such that only those with MAPQ scores higher than or equal to 10 remained, from which the alignment with the highest MAPQ score was retained. Where MAPQ scores for multiple valid alignments were equal, one alignment was randomly selected. Alignments consisting of only one read in a pair were discarded. Unique and multiple alignments in BAM format were combined, and coverage was calculated for each coordinate in the genome using Rsamtools (version 1.26.1). Coverage was normalized by the sum of coverage for each library. The log_2_ ratio of ChIP:input coverage was calculated to control for background and variation in mappability across genomic loci. A library for Columbia genomic DNA (gDNA) that had been extracted using CTAB, fragmented using dsDNA shearase, and subjected to paired-end sequencing on an Illumina NextSEq 500 instrument as described (25, 58) was aligned to TAIR10 and used to calculate log_2_(MNase/gDNA) ratios. Additionally, the first gDNA read in each pair was trimmed to 50 bp, aligned, and used to calculate log_2_(SPO11-1-oligo/gDNA) ratios.

To generate chromosome-scale profiles, mean coverage values within adjacent 10-kb windows were calculated. Log_2_ ratios of windowed ChIP:input coverage were then calculated and smoothed by applying a moving average. Additionally, DNA methylation proportions derived from published bisulfite sequencing reads were used to profile DNA methylation levels at the chromosome scale (61). Spearman’s rank-order correlation coefficients were calculated for each pair of profiled data sets and presented in correlation matrices separately for the chromosome arms and pericentromeres. The pericentromeres are defined as the regions surrounding the centromeres with higher-than-average DNA methylation (26). For analysis along telomere–centromere axes, data values were first calculated in 10-kb windows along the chromosomes. Chromosome arms were then oriented such that each began at the telomere and ended at the centromere and divided into windows along their proportional lengths. Data values were then averaged across all chromosome arms and plotted.

Fine-scale coverage profiles around TAIR10 representative gene transcription start and termination sites (TSSs and TTSs) were generated using the normalizeToMatrix function from the Bioconductor package EnrichedHeatmap (version 1.11.1) (62). Each feature was divided into proportionally scaled windows between start and end coordinates, and 2-kb flanking regions were divided into 20-bp windows. For each window along each feature and its flanking regions, an average value was calculated using the “w0” method for ChIP-seq data. The default profile-smoothing method implemented in the normalizeToMatrix function was applied. The resulting matrix of windowed coverage values was used to generate a mean profile, or a heat map in which each row represents a single feature. Mean profiles and heat maps were plotted such that the distance between feature start and end coordinates along the *x*-axis represents the mean feature length.

### Crossover Mapping via Genotyping by Sequencing.

Wild type and *asy1* Col×Ws and *asy1/+* Col×Ler F_2_ plants were grown, and genomic DNA was extracted from leaf tissue using a CTAB protocol as described (37, 50, 63). A total of 150 ng of DNA was used to generate each sequencing library as described (37, 50, 63). A total of 96 libraries were pooled and sequenced on one lane of an Illumina NextSeq500 instrument using a 300-cycle Mid-Output kit (Illumina). Sequencing data analysis and mapping of crossovers were carried out using the TIGER pipeline as described (33, 37, 50, 63).

### Measuring Crossover Frequency Using Fluorescent FTL Pollen and Seed.

Scoring of fluorescent seeds and measurement of crossover frequency within the *420* genetic interval were performed by microscopy and using CellProfiler as described (39, 64, 65). Scoring of fluorescent pollen grains and measurement of crossover frequency within the *CEN3* FTL genetic interval were performed using an Accuri C2 (BD Biosciences) flow cytometer as described (66). For measurement of crossover interference within the *I3bc* FTL intervals, *qrt1* pollen tetrads were scored using a Leica SP8 confocal microscope. Calculation of crossover frequency and the interference ratio were performed as described (38).

### Cytological Analysis of Meiosis.

Fixation of *Arabidopsis* inflorescences and chromosome spreads of pollen mother cells (PMCs) were performed as described (67). Immunostaining of ASY1, ZYP1, and MLH1 were prepared on acetic acid chromosome spreads using fixed inflorescences. After chromosome spreading, the slides were incubated in boiling 10-mM Tris-sodium citrate, pH 7.0, for 45 s, followed by incubation in 1× PBS with 0.1% Triton X-100 (PBST) for 5 min. Primary antibodies were diluted in a solution of 1% BSA diluted in PBST that was added onto the slides, followed by incubation for 20 h at 4 °C for ASY1 and ZYP1 immunostaining or 40 h at 4 °C for MLH1 immunostaining. The slides were washed in PBST three times for 5 min each at room temperature. Following this, a solution of secondary antibodies diluted in PBST was added, and the slides were incubated for 30 min at 37 °C. The slides were washed in PBST three times for 5 min each at room temperature, and a solution of DAPI/Vectashield was added and a coverslip added to the slide before imaging. The following antibodies were used for immunostaining: α-ASY1 (rat, 1/500 dilution) (11), α-ZYP1 (rabbit, 1/500 dilution) (45), and α-MLH1 (rabbit, 1/200 dilution) (68). Coimmunostaining of ASY1 and H3K9me2 was performed using fresh floral buds. Inflorescences were dissected on damp filter paper under a stereo microscope, and six buds at floral stages 8 to 9 (69) were isolated and transferred to 5 μL of enzyme digestion solution (0.4% cytohelicase, 1.5% sucrose, 1% polyvinylpyrrolidone) on a microscope slide. The buds were dissected to recover the anthers, while the rest of the bud tissue was discarded. The slide was then incubated in a moist box at 37 °C for 1 min, and the anthers were gently opened with a brass rod to release the meiocytes. A total of 5 μL of enzyme digestion solution was added, and the slide was incubated in a moist box at 37 °C for 2 min. After this, 10 μL of 1% Lipsol was added, and the solution was gently mixed with a needle for 1 min before adding 20 μL of 4% paraformaldehyde. The slides were then left to dry for 4 h. Incubation of slides with antibodies for immunostaining of proteins was performed as described earlier. The following antibodies were used: α-ASY1 (rabbit, 1/500 dilution) and H3K9me2 (mouse, 1/100 dilution; Abcam ab1220).

Microscopy was conducted using a DeltaVision Personal DV microscope (Applied Precision/GE Healthcare) equipped with a CCD CoolSNAP HQ2 camera (Photometrics). Image capture was performed using SoftWoRx software version 5.5 (Applied Precision/GE Healthcare). To analyze colocalization of ASY1 and REC8 immunostaining signal on meiotic cells, the contour of chromatin (stained with DAPI) was marked and signal intensity was quantified for every pixel within the marked area using the package coloc2 from Fiji. Following MLH1 immunostaining of diakinesis cells, heterochromatin was identified and marked based on brighter DAPI signal using Fiji (*SI Appendix*, Fig. S5). MLH1 foci were then compared with the marked heterochromatic regions to score overlaps. Synapsed chromosomes immunostained for ZYP1 were marked along their length using the Line Selection Tool of Fiji. Pixel length was recorded and then converted into μM using the Setting Measurement Scale of Fiji. For quantification of ASY1 signal intensity, all slides were prepared alongside one another, and images were captured using the same exposure time. The contour of each cell was marked, and the intensity within this region measured. Each cell was captured as a *Z*-stack of 10 optical sections of 0.2 μM each, and the maximum intensity projection was reconstructed using ImageJ as described (23, 26). A region adjacent to the cell was also marked, and the intensity was measured and used as mean background intensity to subtract from the within-cell intensity.

### Data Availability Statement.

All data are publically available. ASY1 ChIP-seq library data have been deposited in the ArrayExpress database at EMBL-EBI (http://www.ebi.ac.uk/arrayexpress) under accession number E-MTAB-8705 (70). Sequencing data for wild type and *asy1* Col×Ws GBS libraries have been deposited under ArrayExpress accession E-MTAB-8715 (71), and data for *asy1/+* Col×Ler GBS libraries has been deposited under ArrayExpress accession E-MTAB-8725 (72).

## Supplementary Material

Supplementary File

## References

[1] Villeneuve A. M., Hillers K. J., Whence meiosis? Cell 106, 647–650 (2001). 11572770 10.1016/s0092-8674(01)00500-1

[2] Barton N. H., Why sex and recombination? Cold Spring Harb. Symp. Quant. Biol. 74, 187–195 (2009). 19903748 10.1101/sqb.2009.74.030

[3] Mercier R., Mézard C., Jenczewski E., Macaisne N., Grelon M., The molecular biology of meiosis in plants. Annu. Rev. Plant Biol. 66, 297–327 (2015). 25494464 10.1146/annurev-arplant-050213-035923

[4] Vrielynck N., A DNA topoisomerase VI-like complex initiates meiotic recombination. Science 351, 939–943 (2016). 26917763 10.1126/science.aad5196

[5] Berchowitz L. E., Copenhaver G. P., Genetic interference: don’t stand so close to me. Curr. Genomics 11, 91–102 (2010). 20885817 10.2174/138920210790886835 PMC2874225

[6] Zickler D., Kleckner N., Meiotic chromosomes: Integrating structure and function. Annu. Rev. Genet. 33, 603–754 (1999). 10690419 10.1146/annurev.genet.33.1.603

[7] Chelysheva L., AtREC8 and AtSCC3 are essential to the monopolar orientation of the kinetochores during meiosis. J. Cell Sci. 118, 4621–4632 (2005). 16176934 10.1242/jcs.02583

[8] Klein F., A central role for cohesins in sister chromatid cohesion, formation of axial elements, and recombination during yeast meiosis. Cell 98, 91–103 (1999). 10412984 10.1016/S0092-8674(00)80609-1

[9] Ferdous M., Inter-homolog crossing-over and synapsis in Arabidopsis meiosis are dependent on the chromosome axis protein AtASY3. PLoS Genet. 8, e1002507 (2012). 22319460 10.1371/journal.pgen.1002507 PMC3271061

[10] Chambon A., Identification of ASYNAPTIC4, a component of the meiotic chromosome axis. Plant Physiol. 178, 233–246 (2018). 30002256 10.1104/pp.17.01725 PMC6130017

[11] Armstrong S. J., Caryl A. P., Jones G. H., Franklin F. C. H., Asy1, a protein required for meiotic chromosome synapsis, localizes to axis-associated chromatin in Arabidopsis and Brassica. J. Cell Sci. 115, 3645–3655 (2002). 12186950 10.1242/jcs.00048

[12] Osman K., Affinity proteomics reveals extensive phosphorylation of the Brassica chromosome axis protein ASY1 and a network of associated proteins at prophase I of meiosis. Plant J. 93, 17–33 (2018). 29078019 10.1111/tpj.13752 PMC5767750

[13] Kleckner N., Chiasma formation: Chromatin/axis interplay and the role(s) of the synaptonemal complex. Chromosoma 115, 175–194 (2006). 16555016 10.1007/s00412-006-0055-7

[14] Martinez-Perez E., Villeneuve A. M., HTP-1-dependent constraints coordinate homolog pairing and synapsis and promote chiasma formation during C. elegans meiosis. Genes Dev. 19, 2727–2743 (2005). 16291646 10.1101/gad.1338505 PMC1283965

[15] Sanchez-Moran E., Santos J.-L., Jones G. H., Franklin F. C. H., ASY1 mediates AtDMC1-dependent interhomolog recombination during meiosis in Arabidopsis. Genes Dev. 21, 2220–2233 (2007). 17785529 10.1101/gad.439007 PMC1950860

[16] Schwacha A., Kleckner N., Interhomolog bias during meiotic recombination: Meiotic functions promote a highly differentiated interhomolog-only pathway. Cell 90, 1123–1135 (1997). 9323140 10.1016/s0092-8674(00)80378-5

[17] Kim K. P., Sister cohesion and structural axis components mediate homolog bias of meiotic recombination. Cell 143, 924–937 (2010). 21145459 10.1016/j.cell.2010.11.015 PMC3033573

[18] Wojtasz L., Mouse HORMAD1 and HORMAD2, two conserved meiotic chromosomal proteins, are depleted from synapsed chromosome axes with the help of TRIP13 AAA-ATPase. PLoS Genet. 5, e1000702 (2009). 19851446 10.1371/journal.pgen.1000702 PMC2758600

[19] Kim Y., The chromosome axis controls meiotic events through a hierarchical assembly of HORMA domain proteins. Dev. Cell 31, 487–502 (2014). 25446517 10.1016/j.devcel.2014.09.013 PMC4254552

[20] Daniel K., Meiotic homologue alignment and its quality surveillance are controlled by mouse HORMAD1. Nat. Cell Biol. 13, 599–610 (2011). 21478856 10.1038/ncb2213 PMC3087846

[21] Panizza S., Spo11-accessory proteins link double-strand break sites to the chromosome axis in early meiotic recombination. Cell 146, 372–383 (2011). 21816273 10.1016/j.cell.2011.07.003

[22] Goodyer W., HTP-3 links DSB formation with homolog pairing and crossing over during C. elegans meiosis. Dev. Cell 14, 263–274 (2008). 18267094 10.1016/j.devcel.2007.11.016

[23] Lambing C., Arabidopsis PCH2 mediates meiotic chromosome remodeling and maturation of crossovers. PLoS Genet. 11, e1005372 (2015). 26182244 10.1371/journal.pgen.1005372 PMC4504720

[24] He Y., Genomic features shaping the landscape of meiotic double-strand-break hotspots in maize. Proc. Natl. Acad. Sci. U.S.A. 114, 12231–12236 (2017). 29087335 10.1073/pnas.1713225114 PMC5699076

[25] Choi K., Nucleosomes and DNA methylation shape meiotic DSB frequency in *Arabidopsis thaliana* transposons and gene regulatory regions. Genome Res. 28, 532–546 (2018). 29530928 10.1101/gr.225599.117 PMC5880243

[26] Underwood C. J., Epigenetic activation of meiotic recombination near *Arabidopsis thaliana* centromeres via loss of H3K9me2 and non-CG DNA methylation. Genome Res. 28, 519–531 (2018). 29530927 10.1101/gr.227116.117 PMC5880242

[27] Choulet F., Structural and functional partitioning of bread wheat chromosome 3B. Science 345, 1249721 (2014). 25035497 10.1126/science.1249721

[28] Demirci S., Distribution, position and genomic characteristics of crossovers in tomato recombinant inbred lines derived from an interspecific cross between *Solanum lycopersicum* and *Solanum pimpinellifolium*. Plant J. 89, 554–564 (2017). 27797425 10.1111/tpj.13406

[29] Luo C., Li X., Zhang Q., Yan J., Single gametophyte sequencing reveals that crossover events differ between sexes in maize. Nat. Commun. 10, 785 (2019). 30770831 10.1038/s41467-019-08786-x PMC6377631

[30] Lambing C., Interacting genomic landscapes of REC8-cohesin, chromatin, and meiotic recombination in Arabidopsis. Plant Cell 32, 1218–1239 (2020). 32024691 10.1105/tpc.19.00866 PMC7145502

[31] Armstrong S. J., Franklin F. C., Jones G. H., Nucleolus-associated telomere clustering and pairing precede meiotic chromosome synapsis in Arabidopsis thaliana. J. Cell Sci. 114, 4207–4217 (2001). 11739653 10.1242/jcs.114.23.4207

[32] De Muyt A., A high throughput genetic screen identifies new early meiotic recombination functions in Arabidopsis thaliana. PLoS Genet. 5, e1000654 (2009). 19763177 10.1371/journal.pgen.1000654 PMC2735182

[33] Rowan B. A., Patel V., Weigel D., Schneeberger K., Rapid and inexpensive whole-genome genotyping-by-sequencing for crossover localization and fine-scale genetic mapping. G3 (Bethesda) 5, 385–398 (2015). 25585881 10.1534/g3.114.016501 PMC4349092

[34] Sanchez Moran E., Armstrong S. J., Santos J. L., Franklin F. C. H., Jones G. H., Chiasma formation in Arabidopsis thaliana accession Wassileskija and in two meiotic mutants. Chromosome Res. 9, 121–128 (2001). 11321367 10.1023/a:1009278902994

[35] Rabanal F. A., Epistatic and allelic interactions control expression of ribosomal RNA gene clusters in Arabidopsis thaliana. Genome Biol. 18, 75 (2017). 28464948 10.1186/s13059-017-1209-z PMC5414317

[36] Tucker S., Vitins A., Pikaard C. S., Nucleolar dominance and ribosomal RNA gene silencing. Curr. Opin. Cell Biol. 22, 351–356 (2010). 20392622 10.1016/j.ceb.2010.03.009 PMC2912983

[37] Serra H., Massive crossover elevation via combination of *HEI10* and *recq4a recq4b* during *Arabidopsis* meiosis. Proc. Natl. Acad. Sci. U.S.A. 115, 2437–2442 (2018). 29463699 10.1073/pnas.1713071115 PMC5877939

[38] Berchowitz L. E., Copenhaver G. P., Fluorescent Arabidopsis tetrads: A visual assay for quickly developing large crossover and crossover interference data sets. Nat. Protoc. 3, 41–50 (2008). 18193020 10.1038/nprot.2007.491

[39] Melamed-Bessudo C., Yehuda E., Stuitje A. R., Levy A. A., A new seed-based assay for meiotic recombination in Arabidopsis thaliana. Plant J. 43, 458–466 (2005). 16045480 10.1111/j.1365-313X.2005.02466.x

[40] Francis K. E., Pollen tetrad-based visual assay for meiotic recombination in Arabidopsis. Proc. Natl. Acad. Sci. U.S.A. 104, 3913–3918 (2007). 17360452 10.1073/pnas.0608936104 PMC1805420

[41] Wu G., Rossidivito G., Hu T., Berlyand Y., Poethig R. S., Traffic lines: New tools for genetic analysis in Arabidopsis thaliana. Genetics 200, 35–45 (2015). 25711279 10.1534/genetics.114.173435 PMC4423376

[42] Cai X., Dong F., Edelmann R. E., Makaroff C. A., The Arabidopsis SYN1 cohesin protein is required for sister chromatid arm cohesion and homologous chromosome pairing. J. Cell Sci. 116, 2999–3007 (2003). 12783989 10.1242/jcs.00601

[43] Bhatt A. M., The DIF1 gene of Arabidopsis is required for meiotic chromosome segregation and belongs to the REC8/RAD21 cohesin gene family. Plant J. 19, 463–472 (1999). 10504568 10.1046/j.1365-313x.1999.00548.x

[44] Stroud H., Non-CG methylation patterns shape the epigenetic landscape in Arabidopsis. Nat. Struct. Mol. Biol. 21, 64–72 (2014). 24336224 10.1038/nsmb.2735 PMC4103798

[45] Higgins J. D., Sanchez-Moran E., Armstrong S. J., Jones G. H., Franklin F. C. H., The Arabidopsis synaptonemal complex protein ZYP1 is required for chromosome synapsis and normal fidelity of crossing over. Genes Dev. 19, 2488–2500 (2005). 16230536 10.1101/gad.354705 PMC1257403

[46] Rowan B. A., An ultra high-density *Arabidopsis thaliana* crossover map that refines the influences of structural variation and epigenetic features. Genetics 213, 771–787 (2019). 31527048 10.1534/genetics.119.302406 PMC6827372

[47] Drouaud J., Variation in crossing-over rates across chromosome 4 of Arabidopsis thaliana reveals the presence of meiotic recombination “hot spots”. Genome Res. 16, 106–114 (2006). 16344568 10.1101/gr.4319006 PMC1356134

[48] Séguéla-Arnaud M., Multiple mechanisms limit meiotic crossovers: TOP3α and two BLM homologs antagonize crossovers in parallel to FANCM. Proc. Natl. Acad. Sci. U.S.A. 112, 4713–4718 (2015). 25825745 10.1073/pnas.1423107112 PMC4403193

[49] Cifuentes M., Rivard M., Pereira L., Chelysheva L., Mercier R., Haploid meiosis in Arabidopsis: Double-strand breaks are formed and repaired but without synapsis and crossovers. PLoS One 8, e72431 (2013). 23951324 10.1371/journal.pone.0072431 PMC3737152

[50] Yelina N. E., DNA methylation epigenetically silences crossover hot spots and controls chromosomal domains of meiotic recombination in Arabidopsis. Genes Dev. 29, 2183–2202 (2015). 26494791 10.1101/gad.270876.115 PMC4617981

[51] Yant L., Meiotic adaptation to genome duplication in Arabidopsis arenosa. Curr. Biol. 23, 2151–2156 (2013). 24139735 10.1016/j.cub.2013.08.059 PMC3859316

[52] Bomblies K., Jones G., Franklin C., Zickler D., Kleckner N., The challenge of evolving stable polyploidy: Could an increase in “crossover interference distance” play a central role? Chromosoma 125, 287–300 (2016). 26753761 10.1007/s00412-015-0571-4 PMC4830878

[53] Zhang L., Topoisomerase II mediates meiotic crossover interference. Nature 511, 551–556 (2014). 25043020 10.1038/nature13442 PMC4128387

[54] Libuda D. E., Uzawa S., Meyer B. J., Villeneuve A. M., Meiotic chromosome structures constrain and respond to designation of crossover sites. Nature 502, 703–706 (2013). 24107990 10.1038/nature12577 PMC3920622

[55] Rog O., Köhler S., Dernburg A. F., The synaptonemal complex has liquid crystalline properties and spatially regulates meiotic recombination factors. eLife 6, e21455 (2017). 28045371 10.7554/eLife.21455 PMC5268736

[56] Zhang L., Köhler S., Rillo-Bohn R., Dernburg A. F., A compartmentalized signaling network mediates crossover control in meiosis. eLife 7, e30789 (2018). 29521627 10.7554/eLife.30789 PMC5906097

[57] Lambing C., Choi K., Blackwell A. R., Henderson I. R., Chromatin immunoprecipitation of meiotically expressed proteins from Arabidopsis thaliana flowers. Methods Mol. Biol. 2061, 219–236 (2020). 31583663 10.1007/978-1-4939-9818-0_16

[58] Choi K., Recombination rate heterogeneity within Arabidopsis disease resistance genes. PLoS Genet. 12, e1006179 (2016). 27415776 10.1371/journal.pgen.1006179 PMC4945094

[59] Yelagandula R., The histone variant H2A.W defines heterochromatin and promotes chromatin condensation in Arabidopsis. Cell 158, 98–109 (2014). 24995981 10.1016/j.cell.2014.06.006 PMC4671829

[60] Langmead B., Salzberg S. L., Fast gapped-read alignment with Bowtie 2. Nat. Methods 9, 357–359 (2012). 22388286 10.1038/nmeth.1923 PMC3322381

[61] Stroud H., Greenberg M. V. C., Feng S., Bernatavichute Y. V., Jacobsen S. E., Comprehensive analysis of silencing mutants reveals complex regulation of the Arabidopsis methylome. Cell 152, 352–364 (2013). 23313553 10.1016/j.cell.2012.10.054 PMC3597350

[62] Gu Z., Eils R., Schlesner M., Ishaque N., EnrichedHeatmap: An R/Bioconductor package for comprehensive visualization of genomic signal associations. BMC Genomics 19, 234 (2018). 29618320 10.1186/s12864-018-4625-x PMC5885322

[63] Ziolkowski P. A., Natural variation and dosage of the HEI10 meiotic E3 ligase control *Arabidopsis* crossover recombination. Genes Dev. 31, 306–317 (2017). 28223312 10.1101/gad.295501.116 PMC5358726

[64] Ziolkowski P. A., Juxtaposition of heterozygous and homozygous regions causes reciprocal crossover remodelling via interference during Arabidopsis meiosis. eLife 4, e03708 (2015). 10.7554/eLife.03708 PMC4407271 25815584

[65] Carpenter A. E., CellProfiler: Image analysis software for identifying and quantifying cell phenotypes. Genome Biol. 7, R100 (2006). 17076895 10.1186/gb-2006-7-10-r100 PMC1794559

[66] Yelina N. E., High-throughput analysis of meiotic crossover frequency and interference via flow cytometry of fluorescent pollen in Arabidopsis thaliana. Nat. Protoc. 8, 2119–2134 (2013). 24113785 10.1038/nprot.2013.131

[67] Ross K. J., Fransz P., Jones G. H., A light microscopic atlas of meiosis in Arabidopsis thaliana. Chromosome Res. 4, 507–516 (1996). 8939362 10.1007/BF02261778

[68] Chelysheva L., An easy protocol for studying chromatin and recombination protein dynamics during Arabidopsis thaliana meiosis: Immunodetection of cohesins, histones and MLH1. Cytogenet. Genome Res. 129, 143–153 (2010). 20628250 10.1159/000314096

[69] Armstrong S. J., Jones G. H., Meiotic cytology and chromosome behaviour in wild-type Arabidopsis thaliana. J. Exp. Bot. 54, 1–10 (2003). 12456750 10.1093/jxb/erg034

[70] Lambing C., Kuo P. C., Tock A. J., Topp S. D., Henderson I. R., ChIP-seq of ASY1 and controls on meiotic-stage floral buds of Arabidopsis. ArrayExpress. https://www.ebi.ac.uk/arrayexpress/experiments/E-MTAB-8705/. Deposited 14 January 2020.

[71] Lambing C., Kuo P. C., Tock A. J., Topp S. D., Henderson I. R., Identifying crossover locations in Arabidopsis thaliana wild type and asy1 Col-0 × Ws-4 F2 populations using genotyping-by-sequencing. ArrayExpress. https://www.ebi.ac.uk/arrayexpress/experiments/E-MTAB-8715/. Deposited 16 January 2020.

[72] Lambing C., Kuo P. C., Tock A. J., Topp S. D., Henderson I. R., Identifying crossover locations in Arabidopsis thaliana asy1/+ Col × Ler F2 populations using genotyping-by-sequencing. ArrayExpress. https://www.ebi.ac.uk/arrayexpress/experiments/E-MTAB-8725/. Deposited 17 January 2020.

