# Supplementary material for: ASY1 acts as a dosage-dependent antagonist of telomere-led recombination and mediates crossover interference in Arabidopsis
Source: Proc Natl Acad Sci U S A. 2020 Jun 4;117(24):13647–58. doi: 10.1073/pnas.1921055117 (PMC7306779; doi:10.1073/pnas.1921055117)
Supplement: Supplementary File [file pnas.1921055117.sapp.pdf]

PNAS
www.pnas.org

Supplementary Information for:

ASY1 acts as a gene dosage-dependent antagonist of telomere-led recombination and mediates crossover interference in Arabidopsis

Christophe Lambing, Pallas C. Kuo, Andrew J. Tock, Stephanie D. Topp and Ian R. Henderson

Corresponding author: Ian R. Henderson
Email: irh25@cam.ac.uk

This PDF file includes:

Figures S1 to S7
Tables S1 to S14

www.pnas.org/cgi/doi/10.1073/pnas.1921055117

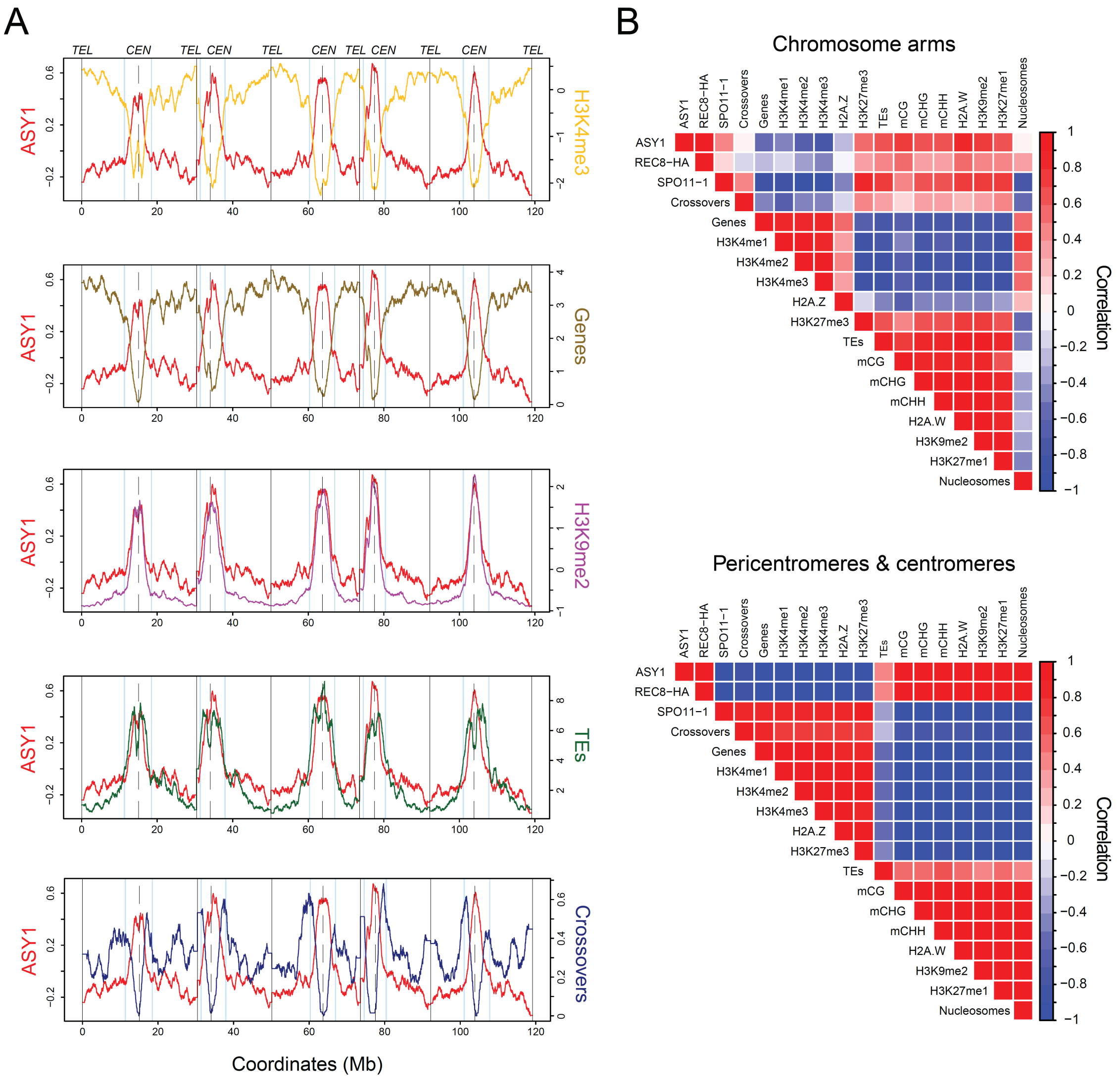

Figure S1. Genome-wide landscape of ASY1, heterochromatin, euchromatin and meiotic recombination. A. Genome-wide profiles of ASY1 (red, $\log_2$(ChIP/input)) compared with H3K4me3 (yellow, $\log_2$(ChIP/input)), genes (brown, genes/10kb), H3K9me2 (purple, $\log_2$(ChIP/input)), transposable elements (dark green, TEs/10kb) and crossovers (dark blue, crossovers/10 kb). **B.** Correlation matrices showing genome-wide Spearman's rank-order correlation coefficients (r_s) for the indicated parameter pairs, with cells color-coded according to the correlation scale shown to the right.

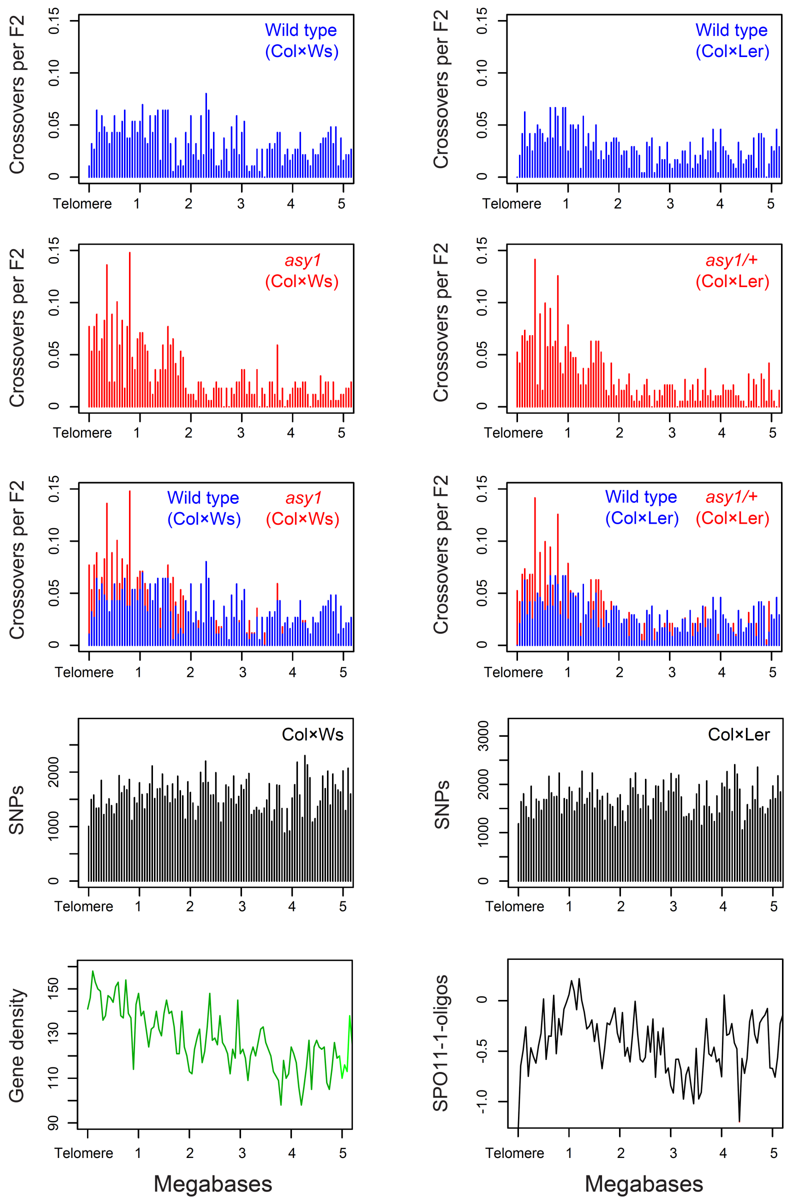

Figure S2. SPO11-1-oligos, SNPs, gene density and crossovers in wild type, *asy1/+* and *asy1* analyzed relative to the nearest telomere. Crossover positions analyzed relative to the closest telomere in wild type Col/Ws (blue) and *asy1* Col/Ws (red), or wild type Col/Ler (blue) and *asy1/+* Col/Ler (red). The lower plots show SNP density (black), gene density (green) or SPO11-1-oligos (black) analyzed over the same regions.

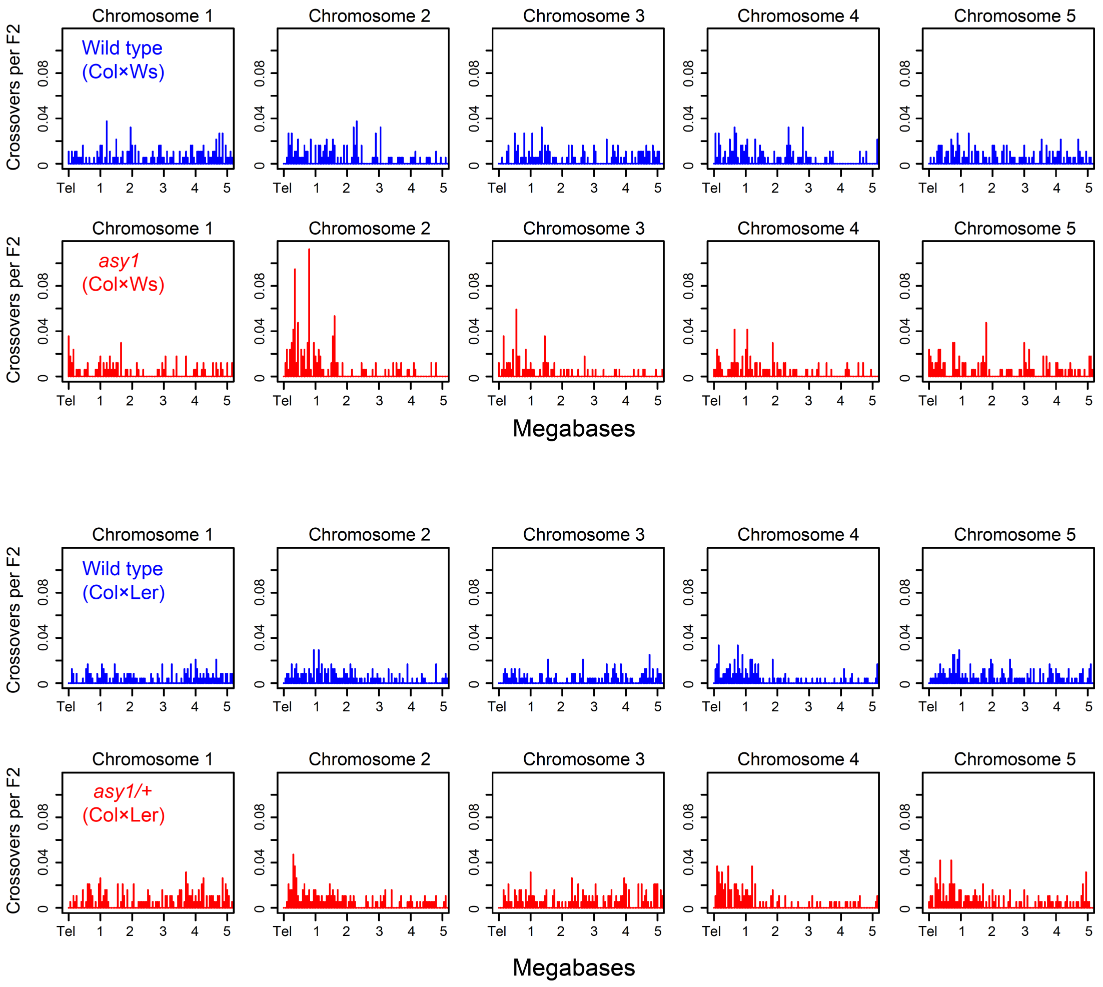

Figure S3. Crossover positions analysed relative to the closest telomere per chromosome. Crossover positions analyzed relative to the closest telomere in wild type Col/Ws (blue) and *asy1* Col/Ws (red), or wild type Col/Ler (blue) and *asy1/+* Col/Ler (red) for each chromosome.

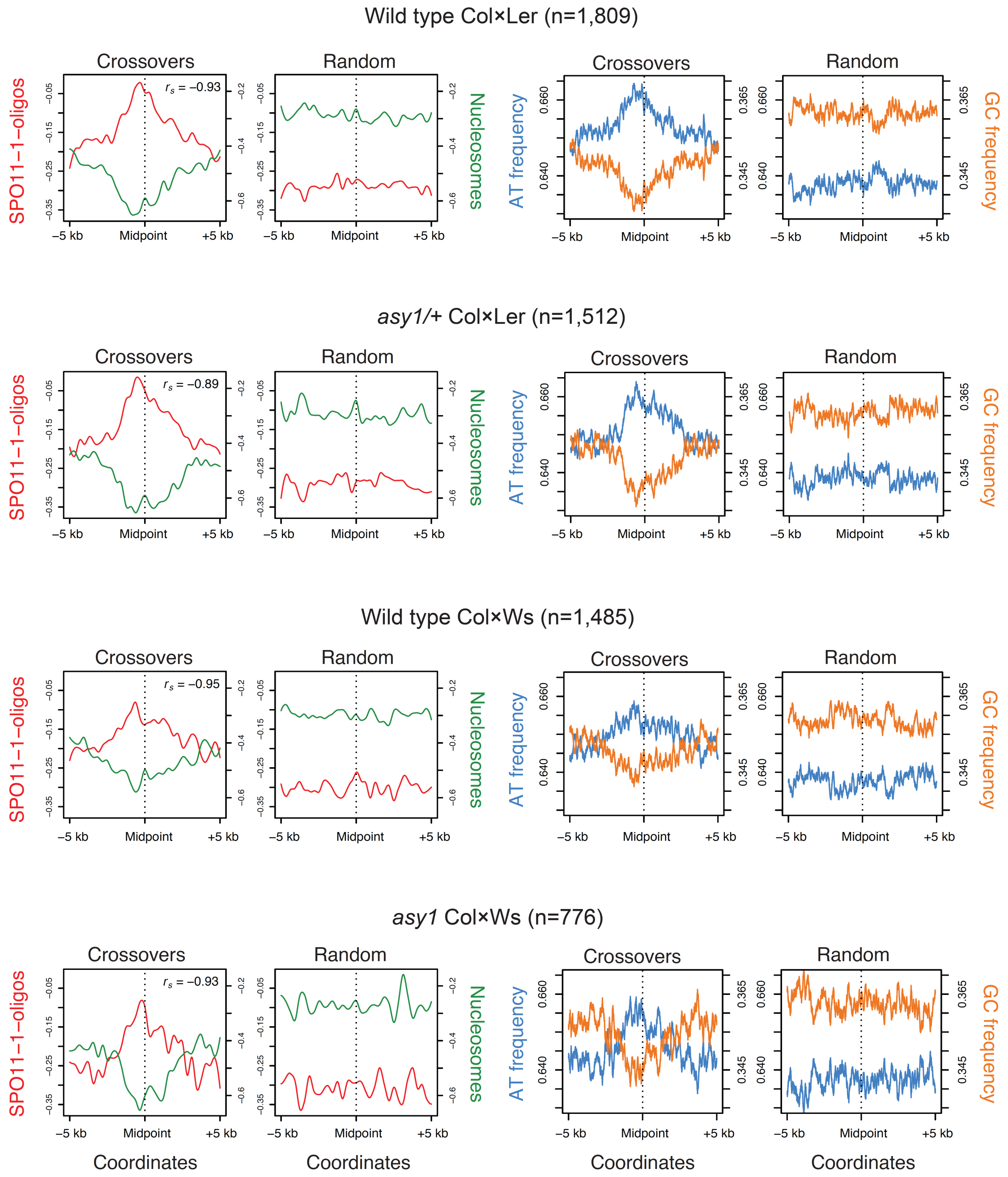

Figure S4. SPO11-1-oligos, nucleosome occupancy and DNA base composition around crossovers in wild type, *asy1/+* and *asy1*. Mean coverage profiles for SPO11-1-oligos (red, log_2(oligos/gDNA)) and nucleosomes (green, log_2(MNase/gDNA)) in 10 kb windows centered on crossover midpoints, identified in wild type Col/Ler, *asy1/+* Col/Ler, wild type Col/Ws and *asy1* Col/Ws, or the same number of random positions of the same widths. For the crossover plots, a correlation value is printed inset for between SPO11-1-oligos and nucleosomes value. Plots are repeated analyzing DNA base frequencies (AT=blue; GC=orange) across the same regions.

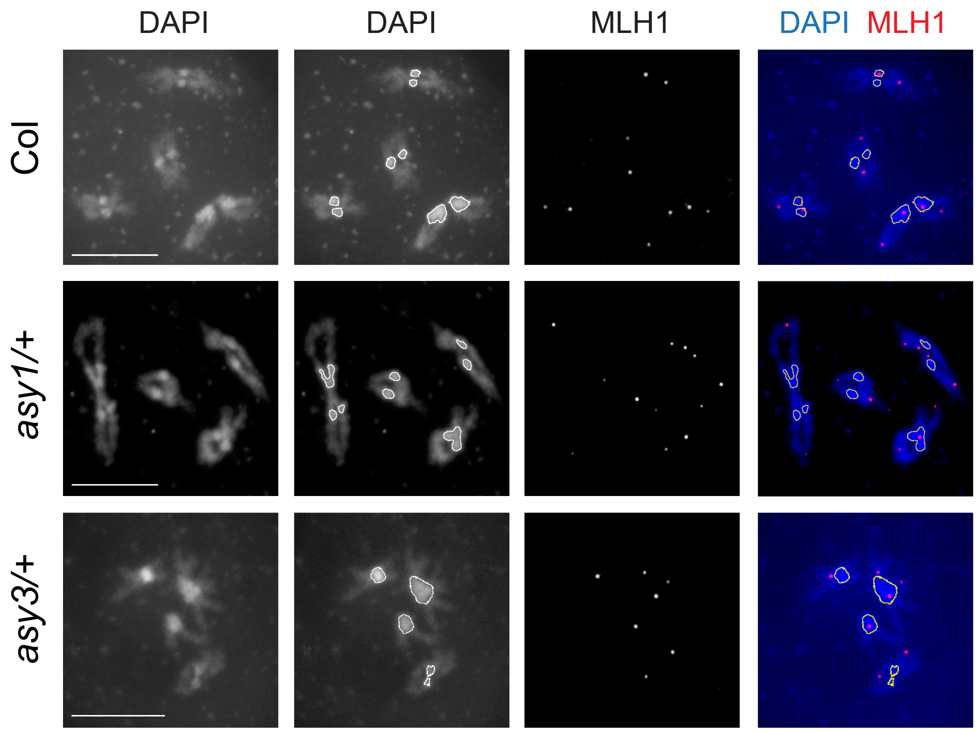

Figure S5. Immunostaining of MLH1 on pollen mother cells and analysis of euchromatin and heterochromatin. Representative images of pollen mother cells immunostained for MLH1 (red) at diakinesis stage in wild type (Col), *asy1-4/+* and *asy3-1/+.* Chromatin was stained with DAPI (blue). The first column show DAPI staining alone. The second column shows DAPI staining, with the heterochromatic regions marked. The third column shows MLH1 immunostaining. The last column shows merged images. Scale bar = 10 μM.

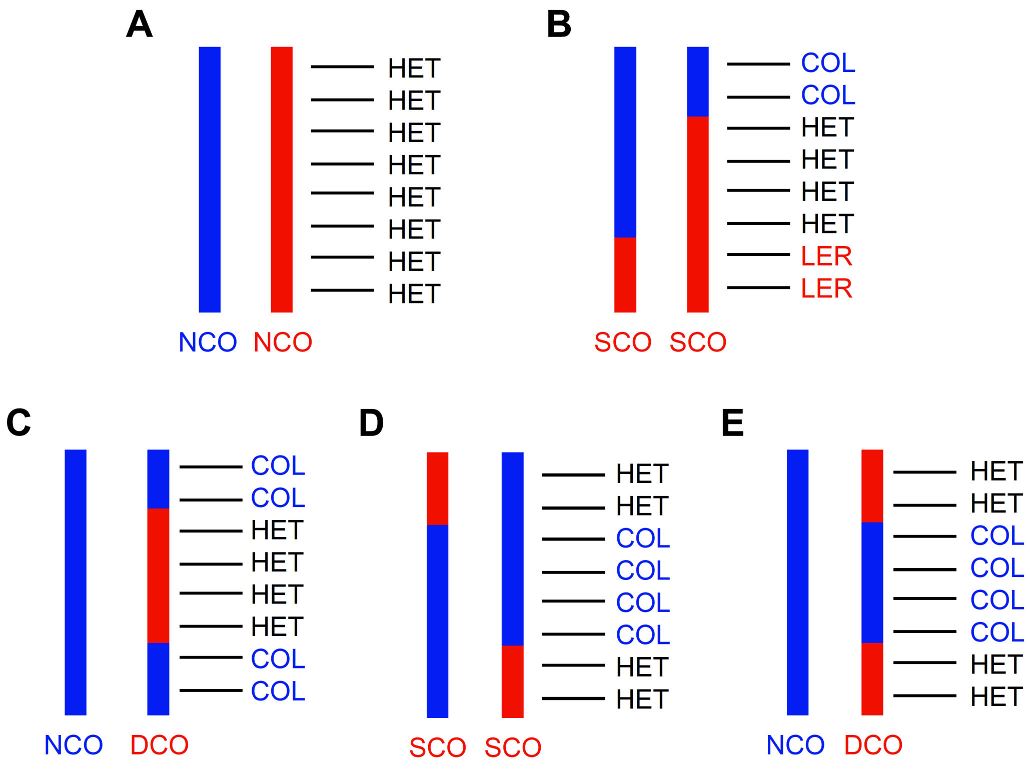

Figure S6. Detection of *cis* Double Crossovers in F_2 sequencing data. A-E. Non-crossover (NCO), single crossover (SCO) and double crossovers (DCO) chromosomes in hypothetical F_2 individuals are shown, colored according to genotype (red=Ler or blue=Col). Presented alongside is a column of observed F_2 genotypes (COL, HET or LER), matching each chromosome figure. Individuals showing the genotype transition HET-COL-HET (D and E) may reflect inheritance of two SCOs, or a *cis* DCO on the same chromatid. COL-HET-COL transitions (C) can only be generated by a true *cis* DCO.

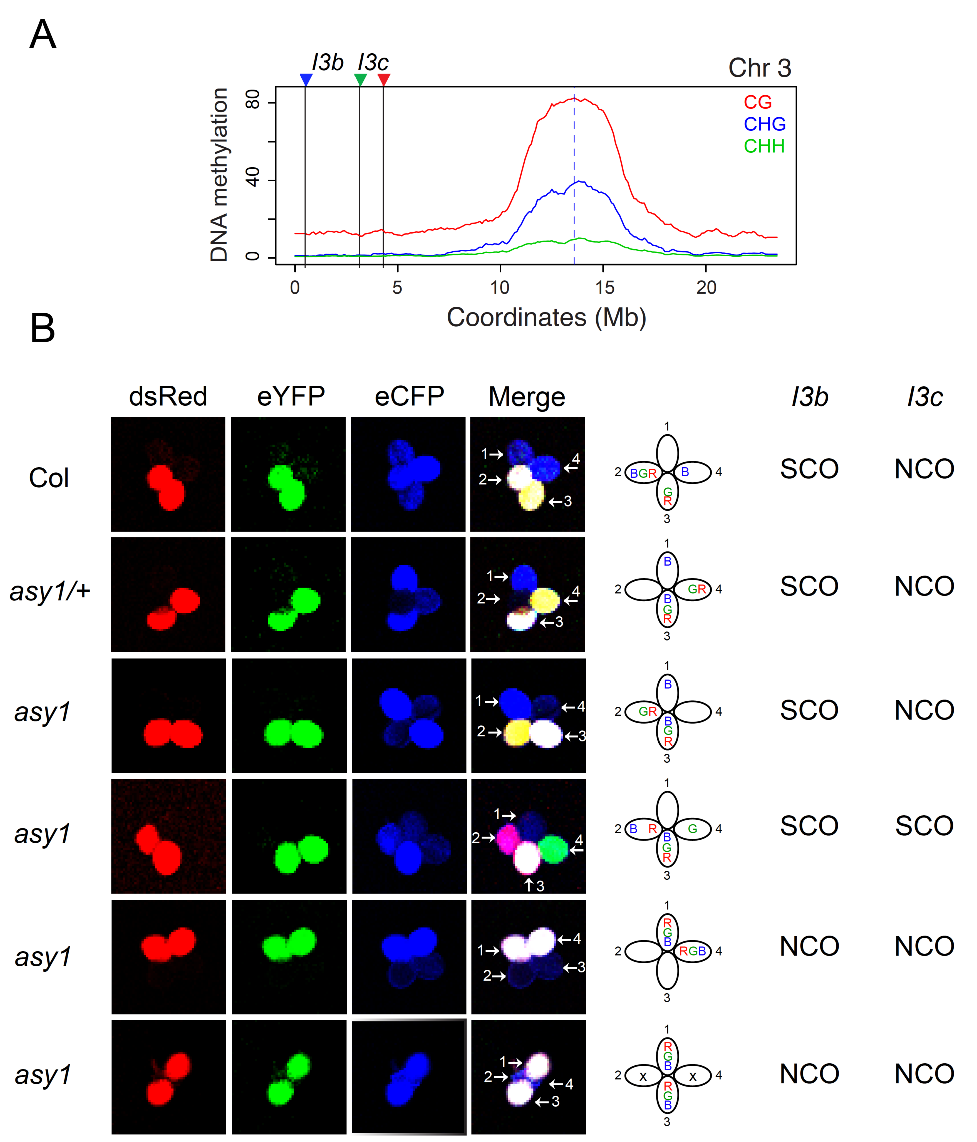

Figure S7. Measurement of crossovers using FTL pollen tetrads in wild type, *asy1/+* and *asy1*. A. DNA methylation (CG, CHG, CHH) in wild type (Col) is plotted along chromosome 3 and the positions of the *I3bc* FTL T-DNAs are indicated by vertical lines and colored triangles. **B.** Fluorescent micrographs of individual FTL tetrads from *I3bc*/++ hemizygotes in wild type (Col), *asy1/+* and *asy1*. Each tetrad is shown imaged for dsRed, eYFP and eCFP fluorescence individually, together with a merged image. The merged image shows a numerical assignment to each tetrad member. These numbers correspond to the tetrad diagrams shown to the right that indicate FTL inheritance patterns. Each tetrad is classified according to *I3b* and *I3c* single crossover (SCO) and non-crossover (NCO) patterns. Note that the final *asy1* tetrad shows the presence of two aborted pollen grains. Aborted pollen grains are represented on the tetrad diagram by “X”.

Table S1. Aligned chromatin immunoprecipitation sequencing (ChIP-seq) reads from ASY1 libraries. Libraries were sequenced using 2×76 base pair reads. Deduplicated read pairs were aligned to the TAIR10 reference genome using Bowtie2 (Version 2.2.9). Uniquely and multiply aligning reads with more than 2 mismatches, and multiply aligning reads with MAPQ scores lower than 10 were discarded. The combined uniquely and multiply aligned reads used for analysis are listed in the 'Final Reads' column.

Library	Total read pairs	Deduplicated	Aligned	Unique (mismatch filtered)	Multiple (mismatch and MAPQ filtered)	Final Reads	Mapped Depth
ASY1 Rep1 ChIP (Col)	47,148,763	29,413,331	26,488,565	13,313,619	2,210,058	15,523,677	17.5×
ASY1 Rep2 ChIP (Col)	57,307,688	42,487,885	39,593,737	21,266,111	3,778,025	25,044,136	28.2×
Pre-immune control (Col)	8,917,585	6,502,968	25,247	8,631	1,723	10,354	0.01×
Leaf control (Col)	5,848,624	3,014,434	8,840	2,786	445	3,231	0.004×

Table S2. Correlations between ASY1 and REC8 ChIP-seq data at varying physical scales. For each ChIP and input library, average library size-normalized coverage values were calculated in adjacent windows of the indicated physical sizes. Log_2(ChIP/input) ratios of windowed coverage values were calculated for each chromosome. Genome-wide Spearman's rank-order correlation coefficients were then calculated for ASY1 and REC8 libraries at varying physical scales.

Library 1	ASY1 Rep1	ASY1 Rep1	ASY1 Rep1	ASY1 Rep2	ASY1 Rep2	REC8-HA Rep1
Library 2	ASY1 Rep2	REC8-HA Rep1	REC8-HA Rep2	REC8-HA Rep1	REC8-HA Rep2	REC8-HA Rep2
2 kb	0.83	0.79	0.80	0.89	0.89	0.86
5 kb	0.88	0.85	0.85	0.92	0.92	0.90
10 kb	0.91	0.88	0.88	0.93	0.93	0.92
20 kb	0.93	0.90	0.90	0.94	0.94	0.94
50 kb	0.95	0.92	0.92	0.95	0.95	0.95
100 kb	0.96	0.93	0.93	0.95	0.95	0.96

Table S3. Crossovers mapped by sequencing Col×Ws wild type and *asy1* and Col×Ler wild type and *asy1/+* F_2 populations. The genotype and cross for different populations is listed, along with the number of crossovers identified from sequencing data for each chromosome and in total. The number of F_2 individuals sequenced in each population and the average number of crossovers per F_2 individual are indicated.

Cross	Genotype	Chr 1	Chr 2	Chr 3	Chr 4	Chr 5	Total	F_2	Crossovers/F_2
Col×Ws	Wild type	379	265	277	232	332	1,485	187	7.94
Col×Ws	*asy1*	139	230	126	122	159	776	169	4.59
Col×Ler	Wild type	434	320	338	303	414	1,809	240	7.54
Col×Ler	*asy1/+*	354	272	289	250	347	1,512	191	7.92

Table S4. Telomeric regions show significantly greater crossovers in *asy1/+* and *asy1* compared to wild type. Crossovers were identified in the indicated F_2 populations and assigned a value based on their distance to the nearest telomere. Crossovers were counted in the indicated 500 kb windows of increasing distance from the telomere. The number of crossovers within and outside each window was calculated, and these values used to perform chi-square tests between wild type and either *asy1/+* or *asy1*. *P* values were adjusted for multiple testing using the Bonferroni method.

	Wild type (Col×Ler)		*asy1/+* (Col×Ler)		
Distance from telomere	Observed crossovers	Non-crossovers	Observed crossovers	Non-crossovers	*P*
0	86	1,277	131	694	$1.22×10^{-11}$
500,000	121	1,242	123	702	$3.46×10^{-4}$
1,000,000	92	1,271	83	742	0.130
1,500,000	73	1,290	69	756	0.133
2,000,000	53	1,310	30	795	1
2,500,000	52	1,311	28	797	1
3,000,000	41	1,322	26	799	1
3,500,000	58	1,305	27	798	1
4,000,000	55	1,308	25	800	1
4,500,000	57	1,306	36	789	1
5,000,000	74	1,289	28	797	0.669
5,500,000	73	1,290	17	808	$4.71×10^{-3}$
6,000,000	79	1,284	27	798	0.188
6,500,000	99	1,264	26	799	$1.59×10^{-3}$
7,000,000	96	1,267	33	792	0.082
7,500,000	102	1,261	34	791	0.039
8,000,000	79	1,284	41	784	1
8,500,000	73	1,290	41	784	1
	Wild type (Col×Ws)		*asy1* (Col×Ws)		
Distance from telomere	Observed crossovers	Non-crossovers	Observed crossovers	Non-crossovers	*P*
0	75	1,111	126	574	$6.82×10^{-11}$
500,000	91	1,095	111	589	$7.85×10^{-7}$
1,000,000	93	1,093	75	625	0.758
1,500,000	59	1,127	74	626	$1.26×10^{-4}$
2,000,000	79	1,107	23	677	0.045
2,500,000	49	1,137	26	674	1
3,000,000	36	1,150	33	667	1
3,500,000	52	1,134	27	673	1
4,000,000	44	1,142	27	673	1
4,500,000	65	1,121	25	675	1
5,000,000	52	1,134	19	681	1

5,500,000	78	1,108	11	689	2.34×10^{-5}
6,000,000	62	1,124	9	691	4.40×10^{-4}
6,500,000	73	1,113	19	681	0.021
7,000,000	89	1,097	25	675	0.014
7,500,000	67	1,119	24	676	0.704
8,000,000	69	1,117	28	672	1
8,500,000	53	1,133	18	682	0.889

Table S5. FTL interval *420* fluorescent seed count data and genetic distance for wild type*, rec8/+, asy1/+, asy1, asy3/+* and *asy3* mutants. Seeds were scored for green and red fluorescence signals using microscopy. Genetic distance (cM) was calculated as $100\times(1-(1-2(N_G+N_r)/N_T)^{1/2})$, where N_G is the number of green alone seeds, N_R is the number of red alone and N_T is the total number of seeds analyzed. All genetic backgrounds were Col×Col.

Individual	Green	Red	Both	None	Total	cM
Wild type	127	127	1032	227	1513	18.5
Wild type	107	116	1059	254	1536	15.76
Wild type	83	83	741	161	1068	16.99
Wild type	134	110	1089	282	1615	16.46
Wild type	122	137	1021	257	1537	18.58
Wild type	119	151	1165	289	1724	17.13
Wild type	109	110	987	242	1448	16.48
Wild type	138	142	1158	313	1751	17.53
Wild type	159	156	1138	285	1738	20.16
Wild type	97	87	796	188	1168	17.24
Wild type	149	147	1154	341	1791	18.18
Wild type	101	99	884	216	1300	16.79
Wild type	127	135	1121	282	1665	17.22
Wild type	143	142	1173	287	1745	17.94
Wild type	154	142	1341	311	1948	16.57
rec8-1/+	166	152	1265	306	1889	18.56
rec8-1/+	199	215	1519	346	2279	20.21
rec8-1/+	83	74	729	177	1063	16.06
rec8-1/+	178	184	1506	403	2271	17.47
rec8-1/+	169	165	1326	366	2026	18.13
rec8-1/+	137	176	1114	281	1708	20.41
rec8-1/+	102	96	857	197	1252	17.31
rec8-1/+	68	70	666	173	977	15.29
rec8-1/+	139	109	1089	280	1617	16.74
rec8-1/+	117	118	994	275	1504	17.08
rec8-1/+	147	124	1121	287	1679	17.71
rec8-1/+	111	114	963	266	1454	16.9
rec8-1/+	156	153	1113	302	1721	19.73
rec8-1/+	122	120	964	281	1487	17.87
rec8-1/+	98	107	770	184	1156	19.35
rec8-3/+	141	135	1209	310	1795	16.78
rec8-3/+	158	137	1301	327	1923	16.74
rec8-3/+	107	102	863	228	1300	17.63
rec8-3/+	105	84	834	220	1243	16.58

rec8-3/+	166	152	1242	342	1902	18.41
rec8-3/+	107	116	848	216	1287	19.16
rec8-3/+	157	158	1039	284	1638	21.55
rec8-3/+	125	145	1189	301	1760	16.74
asy1-1/+	153	155	886	196	1390	25.38
asy1-1/+	164	170	1054	257	1645	22.93
asy1-1/+	187	196	1127	235	1745	25.1
asy1-1/+	170	173	1138	246	1727	22.36
asy1-1/+	150	153	892	189	1384	25.02
asy1-1/+	158	170	1030	251	1609	23.04
asy1-1/+	152	153	883	178	1366	25.61
asy1-1/+	160	183	1100	247	1690	22.92
asy1-1/+	190	184	1085	251	1710	25
asy1-4/+	159	163	1191	294	1807	19.77
asy1-4/+	123	122	882	214	1341	20.34
asy1-4/+	193	193	1337	327	2050	21.04
asy1-4/+	143	159	999	257	1558	21.75
asy1-4/+	107	85	675	163	1030	20.81
asy1-4/+	125	127	954	234	1440	19.38
asy1-4/+	129	148	1011	227	1515	20.36
asy1-4/+	172	164	1131	304	1771	21.22
asy1-4/+	89	102	671	155	1017	20.98
asy1-4/+	193	171	1177	302	1843	22.22
asy1-4/+	133	142	1004	262	1541	19.81
asy1-4	82	79	823	226	1210	14.33
asy1-4	77	60	746	198	1081	13.6
asy1-4	67	54	660	183	964	13.46
asy1-4	93	95	1070	274	1532	13.13
asy1-4	56	62	990	300	1408	8.76
asy1-4	50	59	784	240	1133	10.13
asy1-4	37	43	628	165	873	9.63
asy1-4	62	71	828	256	1217	11.6
asy3-1/+	134	128	1007	237	1506	19.25
asy3-1/+	140	119	860	218	1337	21.73
asy3-1/+	106	113	742	189	1150	21.23
asy3-1/+	183	185	1232	287	1887	21.9
asy3-1/+	133	129	1005	257	1524	19
asy3-1/+	197	156	1226	273	1852	21.34
asy3-1/+	156	138	1020	236	1550	21.22
asy3-1/+	158	176	1270	289	1893	19.56
asy3-1/+	203	161	1277	332	1973	20.56

asy3-1/+	173	144	1119	285	1721	20.53
asy3-2/+	236	233	1563	361	2393	22.02
asy3-2/+	235	256	1548	396	2435	22.75
asy3-2/+	248	241	1554	373	2416	22.85
asy3-2/+	149	154	1103	264	1670	20.18
asy3-2/+	245	263	1572	392	2472	23.25
asy3-2/+	276	268	1802	438	2784	21.95
asy3-2/+	229	243	1627	395	2494	21.17
asy3-2/+	194	242	1565	361	2362	20.58
asy3-2/+	221	217	1422	366	2226	22.12
asy3-2/+	221	238	1574	405	2438	21.04
asy3-1	45	40	798	201	1084	8.18
asy3-1	61	54	757	238	1110	10.96
asy3-1	54	52	865	237	1208	9.2
asy3-1	49	58	721	211	1039	10.89
asy3-1	39	39	937	261	1276	6.31
asy3-1	58	54	899	252	1263	9.3
asy3-1	62	54	949	283	1348	9.01
asy3-1	74	61	659	166	960	15.22
asy3-1	62	77	1067	310	1516	9.63
asy3-1	74	65	919	280	1338	10.99

Table S6. FTL interval *CEN3* fluorescent pollen count data for wild type, *rec8/+*, *asy1/+*, *asy1*, *asy3/+* and *asy3*. Pollen grains were scored for red and green fluorescence using a flow cytometer. Genetic distance (cM) was calculated as $100\times(N_{eYFP}/(N_{eYFP}+N_{both}))$ where N_{eYFP} is the number of green alone pollen grains and N_{both} is the number of pollen grains with both green and red fluorescence.

Genotype	N_{eYFP}	N_{both}	Total	cM
Wild type	731	5353	6084	12.02
Wild type	597	4000	4597	12.99
Wild type	709	5138	5847	12.13
Wild type	456	3177	3633	12.55
Wild type	476	3138	3614	13.17
Wild type	775	5884	6659	11.64
asy1-4/+	184	4016	4200	4.38
asy1-4/+	250	5113	5363	4.66
asy1-4/+	149	2900	3049	4.89
asy1-4/+	160	2600	2760	5.8
asy1-4/+	170	3206	3376	5.04
asy1-4	0	3028	3028	0
asy1-4	0	3010	3010	0
asy1-4	0	3055	3055	0
asy1-4	2	2048	2050	0.1
asy1-4	13	3189	3202	0.41
asy1-4	11	3518	3529	0.31
asy1-4	22	3476	3498	0.63
asy1-4	41	3552	3593	1.14
asy1-4	9	3073	3082	0.29
asy1-4	38	3469	3507	1.08
asy3-1/+	345	3430	3775	9.14
asy3-1/+	541	5683	6224	8.69
asy3-1/+	359	3394	3753	9.57
asy3-1/+	264	2886	3150	8.38
asy3-1/+	336	3313	3649	9.21
asy3-1	14	3,002	3,016	0.46
asy3-1	2	3,546	3,548	0.06
asy3-1	4	3,157	3,161	0.13
asy3-1	1	3,177	3,178	0.03
asy3-1	5	3,538	3,543	0.14
asy3-1	12	3,529	3,541	0.34
asy3-1	6	3,215	3,221	0.19
rec8-1/+	758	5336	6094	12.44
rec8-1/+	568	4223	4791	11.86

rec8-1/+	843	6397	7240	11.64
rec8-1/+	447	3275	3722	12.01
rec8-1/+	667	5138	5805	11.49

Table S7. Seed set per silique in Col, *asy1/+* and *asy3/+*. The number of seeds were counted in 8 siliques for each of 5 individual plants per genotype. Differences between mean measurements in wild type and mutants were evaluated using Mann-Whitney-Wilcoxon tests.

Genotype	Plant	Seed per silique								Mean	*P*
		1	2	3	4	5	6	7	8		
Col	1	53	55	53	54	51	60	52	60	54.8	
Col	2	61	55	45	59	64	62	50	48	55.5	
Col	3	60	53	43	57	52	50	54	54	52.9	
Col	4	61	62	55	54	49	53	48	52	54.3	
Col	5	54	55	60	52	53	50	55	50	53.6	n.d.
asy1-4/+	1	62	61	58	60	58	54	52	48	56.6	
asy1-4/+	2	59	66	63	56	61	63	58	45	58.9	
asy1-4/+	3	63	61	58	65	58	63	59	60	60.9	
asy1-4/+	4	56	58	55	55	47	54	55	54	54.3	
asy1-4/+	5	69	58	51	62	66	63	58	53	60.0	6.9×10^{-4}
asy3-1/+	1	66	50	50	49	59	50	59	56	54.9	
asy3-1/+	2	60	45	43	64	68	60	55	54	56.1	
asy3-1/+	3	52	55	50	56	56	56	61	56	55.3	
asy3-1/+	4	64	55	53	52	56	57	55	63	56.9	
asy3-1/+	5	64	49	59	58	65	63	68	51	59.6	0.054

Table S8. Pollen viability measurement using Alexander staining in Col, *asy1*, asy1/+, *asy3* and *asy3/+*. Pollen were treated with Alexander's stain and visualized under a light microscope. Pollen viability was determined based on the staining and shape of the pollen grains.

Genotype	Plant	Viable	Inviable	Total	% viable	% inviable
Col	1	1000	7	1007	99.3	0.7
Col	2	1000	1	1001	99.9	0.1
Col	3	1007	8	1015	99.2	0.8
Col	4	1033	13	1046	98.8	1.2
Col	5	1033	3	1036	99.7	0.3
Col	6	1000	3	1003	99.7	0.3
asy1-4/+	1	1000	3	1003	99.7	0.3
asy1-4/+	2	1007	14	1021	98.6	1.4
asy1-4/+	3	1000	6	1006	99.4	0.6
asy1-4/+	4	1003	17	1020	98.3	1.7
asy1-4/+	5	1015	16	1031	98.4	1.6
asy1-4/+	6	1000	9	1009	99.1	0.9
asy1-4	1	613	398	1011	60.6	39.4
asy1-4	2	537	490	1027	52.3	47.7
asy1-4	3	585	450	1035	56.5	43.5
asy1-4	4	523	485	1008	51.9	48.1
asy3-1/+	1	1000	10	1010	99.0	1.0
asy3-1/+	2	1006	8	1014	99.2	0.8
asy3-1/+	3	1002	17	1019	98.3	1.7
asy3-1/+	4	1009	6	1015	99.4	0.6
asy3-1/+	5	1007	11	1018	98.9	1.1
asy3-1/+	6	1000	8	1008	99.2	0.8
asy3-1	1	604	136	740	81.6	18.4
asy3-1	2	601	162	763	78.8	21.2
asy3-1	3	526	192	718	73.3	26.7
asy3-1	4	579	152	731	79.2	20.8

Table S9. Immunostained MLH1 foci count at diakinesis in wild type*, asy1/+* and *asy3/+.* Meiotic cells at diakinesis stage were immunostained for MLH1, a marker of Class I crossovers. Total MLH1 foci and MLH1 foci overlapping chromocenters (defined by the DAPI-stained dense regions) were scored. Mann-Whitney-Wilcoxon tests were performed to test for significant differences in MLH1 foci counts between wild type, *asy1-4/+* and *asy3-1/+.*

	Total MLH1 foci			MLH1 foci count on chromocenters		
	Wild type	*asy1-4/+*	*asy3-1/+*	Wild type	*asy1-4/+*	*asy3-1/+*
	12	9	8	2	0	1
	11	10	10	2	0	2
	12	11	7	1	1	1
	9	11	11	2	2	2
	10	9	8	1	1	1
	9	12	10	2	2	0
	10	14	8	1	0	1
	9	8	7	0	0	1
	11	10	9	4	1	0
	11	10	7	2	1	2
	9	10	8	3	1	0
	12	7	8	1	0	0
	11	11	7	2	0	0
	12	11	9	3	1	1
	8	13	12	0	2	1
	10	11	7	2	0	0
	9	13	12	1	1	1
	11	12	8	3	0	1
	12	13	8	1	1	1
	12	12	8	4	2	2
	10	12	7	1	1	1
	10	11	10	1	0	1
	11	13	8	1	0	1
	9	12	8	2	0	1
	10	10	8	0	0	2
	11	13	10	2	0	0
	9	9	8	2	1	1
	13	8	9	4	0	0
	10	10	7	3	2	2
	9	12	11	2	2	0
	9		9	1		0
	11		7	0		4
	10		7	1		2
	9		10	1		1
	10		9	2		1
	9			1		
	10			3		
	10			2		
	8			1		
	11			2		
	10			1		
	10			0		
	12			1		
	11			4		
	13			1		
	12			1		
	12			3		
	12			2		
	11			3		

	10			2		
	9			2		
Mean	10.4	10.9	8.6	1.7	0.7	1.0
SD	1.3	1.7	1.5	1.1	0.8	0.9
P	n.d.	0.128	2.26×10^{-7}	n.d.	4.53×10^{-5}	8.94×10^{-4}

Table S10. ASY1 immunostained signal intensity at leptotene stage in wild type, *asy1/+* and *asy3/+*. ASY1 was immunostained on pollen mother cells at leptotene stage. Images were acquired as Z-stacks of 10 optical sections of 0.2 µM each and total ASY1 signal intensity was measured on the reconstructed maximum intensity projection of the cell using ImageJ. Each intensity was then normalized to the wild type mean intensity value for comparison between samples. A Mann-Whitney-Wilcoxon (MWW) test was used to test for significant differences.

	Wild type	*asy3-1/+*	Wild type	*asy1-4/+*
	1.49	0.56	0.69	1.33
	1.27	0.49	0.68	0.97
	0.88	0.89	1.48	1.15
	0.63	0.91	1.14	0.92
	0.51	1.01	0.69	0.90
	1.02	0.81	0.98	0.38
	0.79	0.70	0.92	0.93
	0.87	0.51	0.65	0.63
	0.88	0.63	1.37	0.41
	0.95	0.71	1.07	0.82
	0.97	0.92	0.70	1.07
	1.71	0.92	0.73	0.47
	0.84	0.77	1.11	0.66
	0.73	0.79	0.97	0.55
	1.00	0.70	1.24	0.64
	0.84	0.71	0.80	0.75
	0.97	0.60	1.28	0.92
	1.11	0.90	1.04	0.69
	0.98		1.06	0.83
	0.87		1.42	0.74
	1.69			
	1.04			
	0.98			
	1.18			
	0.79			
Mean	1.00	0.75	1.00	0.79
SD	0.29	0.15	0.27	0.25
P	n.d.	0.001	n.d.	0.019

Table S11. Synaptonemal complex length at pachytene in wild type, *asy1/+* and *asy3/+*. The synaptonemal complex (SC) was immunostained for ZYP1 and its length (μM) was measured on meiotic cells at pachytene stage. Mann-Whitney-Wilcoxon (MWW) tests were performed to test for significant differences.

	Wild type	*asy1-4/+*	*asy3-1/+*
	192.9	214.1	122.1
	114.9	206.0	212.3
	211.5	168.3	152.7
	181.7	208.7	312.4
	159.9	186.8	177.3
	173.2	202.8	170.0
	215.8	194.7	167.7
	255.0	166.6	126.9
	255.6	181.5	184.8
	199.3	213.3	182.1
	229.1	226.0	213.9
		187.3	
Mean	199.0	196.3	183.8
SD	41.6	18.7	51.7
P	n.d.	0.740	0.270

Table S12. Double crossovers identified in wild type, *asy1*, *asy1/+* and *recq4a recq4b* F_2 populations. Sequencing data was analyzed and regions showing Ler–Het–Ler, Col–Het–Col or Ws–Het–Ws genotype blocks identified in order to find *cis* double crossovers (DCOs). For each cross and genotype the number of DCOs identified is listed, in addition to the mean distance between them, for each chromosome and genome-wide.

Cross	Genotype		Chr 1	Chr 2	Chr 3	Chr 4	Chr 5	Total
Col×Ler	Wild type	DCOs	32	12	14	19	41	118
Col×Ler	Wild type	Distance (Mb)	12.98	9.92	10.76	9.47	11.01	11.16
Col×Ler	*asy1/+*	DCOs	25	11	17	13	32	98
Col×Ler	*asy1/+*	Distance (Mb)	13.43	11.52	11.33	11.02	16.36	13.49
Col×Ler	*recq4a recq4b*	DCOs	138	115	131	85	153	622
Col×Ler	*recq4a recq4b*	Distance (Mb)	4.75	3.76	5.06	4.15	5.10	4.63
Col×Ws	Wild type	DCOs	25	11	13	11	26	86
Col×Ws	Wild type	Distance (Mb)	13.02	9.46	12.10	10.44	12.80	12.03
Col×Ws	*asy1*	DCOs	14	26	11	8	14	73
Col×Ws	*asy1*	Distance (Mb)	10.91	8.04	7.94	7.81	8.99	8.73

Table S13. MLH1 foci counts scored on diakinesis stage bivalents and univalents in wild type and *asy1*. Diakinesis cells were stained with DAPI and immunostained for MLH1. Total MLH1 were counted per cell, in addition to those associated with bivalents or univalents. The total number of bivalents and univalents per cell are listed. The mean and standard deviation (SD) of measurements in each genotype are listed.

Genotype	Total MLH1 foci	MLH1 foci on bivalents	Number of bivalents	MLH1 foci on univalents	Number of univalents
Col	12	12	5	0	0
Col	11	11	5	0	0
Col	12	12	5	0	0
Col	9	9	5	0	0
Col	10	10	5	0	0
Col	9	9	5	0	0
Col	10	10	5	0	0
Col	9	9	5	0	0
Col	11	11	5	0	0
Col	11	11	5	0	0
Col	9	9	5	0	0
Col	12	12	5	0	0
Col	11	11	5	0	0
Col	12	12	5	0	0
Col	8	8	5	0	0
Col	10	10	5	0	0
Col	9	9	5	0	0
Col	11	11	5	0	0
Col	12	12	5	0	0
Col	12	12	5	0	0
Col	10	10	5	0	0
Col	10	10	5	0	0
Col	11	11	5	0	0
Col	9	9	5	0	0
Col	10	10	5	0	0
Col	11	11	5	0	0
Col	9	9	5	0	0
Col	13	13	5	0	0
Col	10	10	5	0	0
Col	9	9	5	0	0
Col	9	9	5	0	0
Col	11	11	5	0	0
Col	10	10	5	0	0
Col	9	9	5	0	0
Col	10	10	5	0	0

Col	9	9	5	0	0
Col	10	10	5	0	0
Col	10	10	5	0	0
Col	8	8	5	0	0
Col	11	11	5	0	0
Col	10	10	5	0	0
Col	10	10	5	0	0
Col	12	12	5	0	0
Col	11	11	5	0	0
Col	13	13	5	0	0
Col	12	12	5	0	0
Col	12	12	5	0	0
Col	12	12	5	0	0
Col	11	11	5	0	0
Col	10	10	5	0	0
Col	9	9	5	0	0
Mean	10.41	10.41	5	0	0
SD	1.27	1.26	0	0	0
asy1	11	5	2	6	6
asy1	9	6	3	3	4
asy1	11	3	1	8	8
asy1	9	8	2	1	6
asy1	9	7	3	2	4
asy1	13	5	2	8	6
asy1	10	3	2	7	6
asy1	12	4	2	8	6
asy1	12	6	2	6	6
asy1	10	7	2	3	6
asy1	8	3	2	5	6
asy1	11	6	2	5	6
asy1	10	3	2	7	6
asy1	11	7	3	4	4
asy1	9	5	2	4	6
asy1	9	2	1	7	8
asy1	11	3	3	8	4
asy1	9	4	2	5	6
asy1	10	6	3	4	4
asy1	10	4	2	6	6
asy1	10	6	3	4	4
asy1	10	10	3	0	4
asy1	13	9	3	4	4

Mean	10.3	5.3	2.26	5	5.48
SD	1.33	5.1	0.62	2.28	1.24

Figure S14. Normalized inter-MLH1 foci distances measured in wild type (Col) and *asy1* bivalents in diakinesis stage meiocytes. Diakinesis stage meiocytes were spread and DNA was stained with DAPI and MLH1 was immunostained, in wild type (Col) and *asy1.* The distance between two MLH1 foci on a single bivalent were measured using imageJ and normalised by the total length of the bivalent. 25 wild type (Col) cells and 23 *asy1* cells were analysed.

Genotype	
Col	*asy1*
21.9	13.3
19.4	50.5
15.8	14.1
33.5	85.2
47.0	59.4
42.7	43.6
23.7	38.8
28.7	12.0
35.3	17.0
28.8	48.4
43.0	20.2
16.4	12.2
15.4	26.6
30.5	5.0
18.2	11.8
41.7	51.3
32.6	9.2
17.8	20.5
80.6	24.2
44.5	30.0
27.5	6.0
36.0	29.0
64.8	12.3
25.4	19.0
74.4	16.7
32.1	9.2
52.9	23.6
25.6	8.4
54.8	59.8
36.0	16.8
26.7	14.4
30.3	11.3
39.6	12.5
33.8	81.3
41.6	62.1
17.2	26.3
33.7	60.1
35.2	26.7
41.0	17.3
50.2	13.7
60.7	30.8
41.4	27.4
25.5	13.9
32.8	30.3

32.8	19.1
30.3	55.2
35.0	20.5
34.0	64.8
54.7	21.9
53.6	29.5
75.3	19.7
57.8	17.5
31.8	24.3
37.5	15.8
41.5	15.7
22.0	29.8
22.0	3.6
17.8	70.2
43.3	25.0
64.7	10.2
40.9	16.3
60.6	11.1
62.6	12.7
68.9	9.9
30.0	56.6
22.3	14.5
30.3	52.9
55.8	13.0
78.9	57.9
50.8	14.1
25.6	
29.9	
33.8	
49.1	
18.6	
37.1	
33.7	
67.7	
35.2	
26.8	
24.9	
36.6	
29.2	
10.2	
41.8	
60.1	
69.4	
21.6	
35.4	
28.6	
24.2	
39.5	
39.1	
44.0	
45.8	
15.0	
24.6	

45.6	
28.2	
27.8	
58.8	
54.2	
22.0	
66.1	
32.6	
29.0	
52.0	
36.9	
26.8	
9.7	
24.9	
68.0	
13.6	
18.1	
38.2	
15.6	
31.8	
4.6	
46.1	
43.4	
27.1	
21.9	
44.8	
55.1	
60.3	
77.3	
10.0	
28.8	
17.1	
43.4	
Mean = 37.4	Mean = 27.5
SD = 16.6	SD = 19.7
